# Metabolic shifts in lipid utilization and reciprocal interactions within the lung metastatic niche of triple-negative breast cancer revealed by spatial multi-omics

**DOI:** 10.1038/s41419-024-07205-4

**Published:** 2024-12-18

**Authors:** Jung-Yu Kan, Hsiao-Chen Lee, Ming-Feng Hou, Hung-Pei Tsai, Shu-Fang Jian, Chao-Yuan Chang, Pei-Hsun Tsai, Yi-Shiuan Lin, Ying-Ming Tsai, Kuan-Li Wu, Yung-Chi Huang, Ya-Ling Hsu

**Affiliations:** 1 Department of Surgery, Kaohsiung Medical University Hospital, Kaohsiung Medical University, Kaohsiung, 80756 Taiwan; 2 Division of Breast Oncology and Surgery, Department of Surgery, Kaohsiung Medical University Hospital, Kaohsiung Medical University, Kaohsiung, 80756 Taiwan; 3 School of Post Baccalaureate Medicine, Kaohsiung Medical University, Kaohsiung, 807 Taiwan; 4 Division of Plastic Surgery, Department of Surgery, Kaohsiung Medical University Hospital, Kaohsiung Medical University, Kaohsiung, 807 Taiwan; 5 Department of Biomedical Science and Environment Biology, Kaohsiung Medical University, Kaohsiung, 807 Taiwan; 6 Division of Neurosurgery, Department of Surgery, Kaohsiung Medical University Hospital, Kaohsiung, Taiwan, ROC; 7 Graduate Institute of Medicine, College of Medicine, Kaohsiung Medical University, Kaohsiung, 807 Taiwan; 8 Division of Pulmonary and Critical Care Medicine, Kaohsiung Medical University Hospital, Kaohsiung Medical University, Kaohsiung, 807 Taiwan; 9 Drug Development and Value Creation Research Center, Kaohsiung Medical University, Kaohsiung, 807 Taiwan; 10 National Pingtung University of Science and Technology, Department of Biological Science and Technology, Pingtung, 912 Taiwan

**Keywords:** Cancer metabolism, Transcriptomics

## Abstract

The Triple-Negative Breast Cancer (TNBC) subtype constitutes 15-20% of breast cancer cases and is associated with the poorest clinical outcomes. Distant metastasis, particularly to the lungs, is a major contributor to the high mortality rates in breast cancer patients. Despite this, there has been a lack of comprehensive insights into the heterogeneity of metastatic tumors and their surrounding ecosystem in the lungs. In this study, we utilized spatial RNA sequencing technology to investigate the heterogeneity of lung metastatic tumors and their microenvironment in two spontaneous lung metastatic mouse models. Our findings revealed an increase in metabolic-related genes within the cancer cells, with the hub gene *Dlat* (Dihydrolipoamide S-Acetyltransferase) showing a significant association with the development of lung metastatic tumors. Upregulation of *Dlat* led to the reprogramming of fatty acid utilization, markedly enhancing the bioenergetic capacity of cancer cells. This finding was corroborated by the increased dependence on fatty acid utilization in lung metastatic cancer cells, and inhibition of *Dlat* in breast cancer cells exhibited a reduced oxygen consumption rate. Consequently, inhibition of Dlat resulted in decreased survival capacity of breast cancer by reducing cancer stem cell properties and cell adhesion in the lung in vivo. The three cell components within the lung metastatic niche, including CD163^+^ macrophages, neutrophils, and endothelial cells, expressed elevated levels of ApoE, leading to the secretion of various protumorigenic molecules that promote cancer cell growth in the lung. These molecules include galectin-1, S100A10, S100A4, and S100A6. Collectively, our findings highlight the lipid metabolism reprogramming of cancer and components of the tumor microenvironment that support lung metastasis of TNBC breast cancer.

## Introduction

Breast cancer (BC) is currently one of malignant and is estimated to account for 30% of all new cancer diagnosed in women. It is also the major cause of most cancer-related deaths, and metastasis of breast cancer to vital organs is considered the principal factor [1–3]. Therefore, BC remains a vital public health concern, particularly triple-negative breast cancer (TNBC), which is the absence of well-defined and universal targets for therapy, resulting in the poorest prognosis among breast cancer patients [4]. The lung is the second most common site of breast cancer metastasis, with a 5-year overall survival of approximately 16.8% [3–5]. The poor clinical outcome of TNBC patients with lung metastasis is attributed to the limited options of therapeutic strategy accompanying inoperable lesions [6]. Improving our understanding of the molecular mechanism of lung metastasis in BC might open novel diagnostic and new therapeutic approaches for amending the prognosis and treatment of advanced breast cancer.

Heterogeneity typically exists between similar tumor types, resulting in subtypes (intertumor heterogeneity) or within tumors of the same type (intratumor heterogeneity). Intratumor heterogeneity endows cancer cells with different oncogenic properties and resistance to treatment; thus, they exhibit different abilities to cope with various challenges, such as new microenvironment adaption during metastasis [7–9]. The tumor microenvironment (TME) is a significant regulator of cancer progression. The components of TME, such as cancer fibroblast populations, tumor vasculature, immune system infiltration, and extracellular matrix remodeling, may cause different tumor heterogeneity [10, 11]. In addition to TME of the primary site, the metastatic niche is also thought to change before/during cancer spreading, and it also has a significant influence on the determination of cancer cell fate when disseminated cancer cells arrive at distant sites [12–14]. Conceivably, the phenotypic difference between cancer cells and distinct TME eventually determined the development of tumor heterogeneity in metastatic niches [15, 16]. Comprehensive profiling of the metastatic lung TME and its influence on breast cancer development has seen few, therefore, systematic and deepened research on the molecular heterogeneity of metastatic breast cancer and lung niche would upgrade the ability to explore more effective metastasis-targeting agents and improve the prognosis of BC patients with lung metastasis.

Spatial transcriptomics (ST) and proteomics technologies (and proteomics), based on either RNA sequencing or imaging, provide an unprecedented opportunity to generate innovative insights into gene regulation in the spatial tissue context. ST offers spatial information and is a powerful tool to characterize genetic and functional heterogeneities [17, 18]. Here, we use ST and multiple-staining imaging systems to explore the heterogeneity of metastatic breast cancer and lung niche using a TNBC animal model. We leverage two spontaneous lung metastatic models, TNBC 4T1 and MMTV-PyVT, to recapitulate spontaneous lung metastasis to investigate the tumor complexity in vivo, and the results reveal that fatty acid utility triggered metabolism-related genes required for lung spreading in breast cancer. Gene enrichment analysis and ligand-receptor pairs coupled to functional approaches implicate macrophage-derived soluble factors contributed to the remodeling of TMEs and tumor aggressiveness. Our work describes a functional interaction between TMEs and the malignant states that manipulate cancer progression during distant metastasis. We propose that increased fatty acid utility acts as a hub transition of cancer cells and immune responses of TME control BC lung metastatic fate.

## Materials and methods

### Cell culture

Breast cancer cell lines Hs578T and 4T1 and human monocytic leukemia cell line THP-1 were obtained from American Type Culture Collection. Breast cancer cell lines were grown in DMEM supplemented with 10% Fetal Bovine Serum (FBS), while THP-1 were cultured in RPMI 1640 (ThermoFisher, USA) medium with 10% FBS. All cell lines authenticated by short tandem repeat analysis. Each cell line was confirmed negative for mycoplasma contamination using mycoplasma test kits (Mycoalert Mycoplasma Detection Kit; Capsugel; Lonza Group, Ltd.) every 3 months. THP-1 monocytes are differentiated into macrophages by treating the cells with 150 nM phorbol 12-myristate 13-acetate (PMA, Sigma, P8139) for 24 h and then incubated in RPMI 1640 medium for an additional 24 h to allow for full differentiation into macrophages. THP-1-derived macrophages and Hs578T cells were co-cultured in a transwell system using RPMI 1640 medium for 16 h. In the blocking experiments, Hs578T cells were treated with or without etomoxir (4 μM) for 24 h. After washing, Hs578T cells were co-cultured with THP-1-derived macrophages, as described above.

### Animal model

All mice procedures related to animal use were conducted and approved in accordance with the Institutional Animal Care and Use Committee at Kaohsiung Medical University, and all animals were treated in accordance with the National Laboratory for Experimental Animals guidelines. BALB/c mice (female, 8-week old) received wild-type, or *Dlat*-knockdown/knockout 4T1 cells implantation (500,000 cells per mammary fat pad), allowing cells to metastasize to the lung for 21 days spontaneously. Female and male MMTV-polyoma middle T antigen (PyMT) transgenic mice on an FVB background (double transgenic; FVB/N-Tg [MMTV-PyVT] 634 Mul/J^−1^) were obtained from Jackson Laboratories (Bar Harbor, ME, USA). A breeding colony was established by crossing FVB/NJ females with hemizygous MMTV-PyVT 634 Mul/J^−1^males. These mice began developing palpable mammary tumors at around 4–5 weeks of age, with lung metastases appearing at approximately 15 weeks. Lungs of mice were harvested after the indicated times, fixed and embedded in paraffin, and sectioned for ST or CODEX ((CO-Detection by indEXing) analysis. For cell sorting, DiR-staining 4T1-L or lung metastatic breast cancer (MMTV-L, isolated from lung of MMTV-PyVT mice) cells were transplanted into BALB/c mice from mammary fat pads or tail vein injection. The animals were then sacrificed on 24 h (cell adhesion), 7 days (cell proliferation), and 21 days (tumor nodules). The ability of cell adhesion and proliferation in the lungs of mice was assessed by counting DiR-staining 4T1 or MMTV-L cells in a frozen section of the lung by a laser scanning confocal microscope (LSM700, Carl Zeiss, Oberkochen, Germany). The 4T1 breast cancer, macrophage cells, and CD11b^+^F4/80^+^ macrophage in the lungs were isolated by mincing, collagenase type I digestion, and sorting using cell sorter BD FACS Melody (BD Biosciences).

### Slide preparation and ST sequencing

Visium Spatial Gene Expression Slides (Visium Spatial Gene Expression Slide Kit) were obtained from 10x Genomics (Pleasanton, CA, USA), and they contained capture areas (6.5 mm^2^) with approximately 5000 barcodes and gene expression spots. The RNA integrity of formalin fixed & paraffin embedded (FFPE) sections were evaluated using the Agilent 4150 TapeStation system. RNA quality. The lung FFPE blocks were cut into 10 mm-thickness tissue blocks and attached to the capture area of slides and then fixed and sequentially stained by Mayer’s hematoxylin (Millipore Sigma, MHS16) for 3 min, bluing buffer (cat no, 6697, Thermo Scientific) for 1 min, and eosin (cat no, HT110116, Millipore Sigma) for 1 min. The FFPE slide images were documented at 20× magnification using a TissueFAX (Tissue Gnostics, Vienna, Austria). The slides were permeabilization, and the mRNA released from lung sections during permeabilization, and the cDNA library was established according to the Visium spatial gene expression user guide. The cDNA library was sequenced on a NextSeq 2000 Systems (Illumina) with a sequencing depth of approximately 250‒270 M reads for each sample. (San Diego, CA, USA).

### Raw sequencing data processing

The Spatial Transcriptomics and bright-field H&E staining images were analyzed and merged by Space Ranger (version 1.1.0, 10x Genomics (Pleasanton, CA, USA), align reads, generate feature-spot matrices, and perform clustering and gene expression analysis. These pipelines combined Visium-specific algorithms with the widely used RNA-seq aligner STAR.

### Weighted gene co-expression network analysis (WGCNA)

The accessible R package of scWGCNA (v0.0.0.9) for ST was used for co-expression analysis (https://github.com/smorabit/hdWGCNA). The Pearson Correlation Coefficient was used to assess similarities between paired mRNAs based on gene expression matrices. These similarities were then converted into adjacency matrices, where a soft-thresholding power was applied to emphasize strong gene connections while minimizing weaker correlations. The adjacency matrix was transformed into a topological overlap measure (TOM) to better capture the strength of connections between genes. Hierarchical clustering was performed using the TOM as input, allowing for the identification of network modules through the DynamicTreeCut method. Modules with high similarity were subsequently merged based on a specified threshold value. The module eigengene expression, adjacency matrix heat-map, and other related parameters were calculated and visualized.

### Bioinformatics analysis of differential expression genes (DEG) analysis, Gene Set Enrichment Analysis (GSEA), and trajectory analysis

Signature genes needed to be expressed, and the changes in gene expression were > 2-fold (log2 FC > 1, *p*-value < 0.05). Pathway enrichment analysis in specific spots was performed using the KEGG database via the DAVID website (https://david.ncifcrf.gov). This revealed key pathways and enriched biological processes related to gene expression in those regions, offering insights into underlying mechanisms (*p* < 0.05). Trajectory analysis was executed using Monocle 2 (v2.22) to determine cell differentiation pathways and progression states. CytoTRACE was utilized to assess the transcriptional diversity of malignant cells based on their differentiation or stemness status (https://cytotrace.stanford.edu). Cells were assigned a CytoTRACE score reflecting their differentiation potential, where higher scores indicate greater stemness and less differentiation. Pathway enrichment analyses were completed using the ssGSEA with default parameters gene sets were acquired from the MSigDB database (https://www.gsea-msigdb.org/gsea/msigdb/).

### Multiplex-CODEX staining and imaging of the lungs of mice

Barcode-conjugated antibody staining of tissue sections mounted on poly-lysine coated coverslips was performed using a commercially available CODEX Staining Kit for FFPE tissue using a commercial Akoya CODEX instrument (Akoya Biosciences, Marlborough, MA) with a Keyence BZ-X800 microscope plus Nikon PlanApo NA 0.75 objective. Images were acquired at 20X magnification and filter cubes for TRITC (550), CY5 (647), and CY7 (750) complementary fluorescent reporters and DAPI (Akoya Bio-sciences). Typical images are 3 × 3 mm, including acquisition of 5 Z-stack images. The images and the quantitation of specific cell types were collected using the CODEX Multiplex Analysis Viewer (MAV) software. The antibodies used in this study are listed in Supplementary Table 1.

### Target genes knockdown or knockout by shRNA or siRNA transfection, and CRISPR/Cas9

4T1 were transfected with control shRNA or *Dlat* shRNA, and the stable clones were established by puromycin selection. The knockdown efficacy of Dlat was confirmed by quantitative real-time reverse transcription polymerase chain reaction assay (qRT-PCR) and Western blot, as described below. 4T1-L and MMTV-L cells (MMTV cells isolated from lung of MMTV-PyVT mice) cells were co-transfected with a Dlat-specific gRNA vector (Cat# KN504604, OriGene Technologies) using the Turbofectin transfection reagent. Two distinct gRNA vectors were employed, containing *Dlat*-targeting sequences 5′-GGGGGATCCCACTACCGCAA-3′ (gRNA1) and 5′- CGCTTGCTGCGACAACTCCT-3′ (gRNA2). Stable clones of both 4T1-L and MMTV-L cells were analyzed by genomic PCR with primers flanking the predicted target sites in the *Dlat* gene. The primers used were CTTTTACGACACTGCGCGAC and TTCAGCAAAGCCACTCCCTT.

ApoE knockdown was performed using Accell siRNA technology (Dharmacon). THP-1 cells were grown in Accell siRNA Delivery Medium and incubated with control or Accell™ siRNA (1 μM, Dharmacon) specifically targeting ApoE. The siRNA was added directly to the culture medium without the need for additional transfection reagents, following the manufacturer’s instructions. Cells were incubated for 48 h to allow for efficient gene silencing. Knockdown efficiency was confirmed via quantitative real-time PCR.

### qRT-PCR

The total RNA of the 4T1 or THP-1 cells was extracted and cDNA was converted form RNA using RT Reagent Kit (RR037A, TaKaRa), and 50 ng cDNA was used with Fast SYBR Green Master Mix (4385612, Applied Biosystems), followed by reactions using a QuantStudio 3 machine (Applied Biosystems). *Gapdh* was used as the internal control gene to give the tested genes a relative fold change using the 2^−ΔΔCt^ method. The qRT-PCR primers are listed in SupplementaryTable 2.

### Western blot

Western blot analysis is used to detect the expression levels of specific proteins. First, cells were collected, and total protein is extracted using a RIPA lysis buffer. Protein concentration is determined using the bicinchoninic acid assay. Total protein was separated by sodium dodecyl sulfate-polyacrylamide gel electrophoresis, followed by transfer onto a polyvinylidene difluoride membrane. The membrane is blocked with 5% milk and then incubated with a primary antibody overnight, followed by incubation with a horseradish peroxidase-conjugated secondary antibody for 1 h. The membrane is then washed, and protein bands are visualized using enhanced chemiluminescence detection reagents and an imaging system. The antibodies are listed in Supplementary Table 1

### Cell functional assays

The cell proliferation of 4T1 or Dlat knockdown was assessed by Premixed WST-1 Cell Proliferation Reagent (Clontech, Mountain View, CA) after incubation 24, 48, and 72 h. The migration of cells was assessed by wound healing and transwell (pore size: 0.8 μm) migration after 24 h incubation. Control plasmid transfected 4T1-L, Dlat knockdown/knockout 4T1-L cells, control plasmid transfected MMTV-L or Dlat-knockout MMTV-L were seeded in a low-attached dish for 14 days, and the feature of cancer stem cells (CSCs) in cancer spheroids was determined by an ALDEFLUOR™ Assay Kit (StemCell technologies, Durham, NC, USA) with a BD Accuri™ flowcytometry (BD Bioscience, San Jose, CA, USA). The cells were stained with DEAB cocktail (DEAB reagent; 5 μl ALDF reagent; 2.5 μl ALDF buffer; 500 μl/test) as the negative control.

### Cell metabolic assay

Control cDNA transfected 4T1-L or Dlat knockdown 4T1-L cells (6 × 10^3^) or breast cancer cells isolated from lungs/primary sites (4T1-L and MMTV-L) or primary sites (4T1-P and MMTV-P) of mice were seeded in XF 96-well and cultured in media in XF HS mini plate. The XF Cell Mito Stress Test kit (Cat no. 103010-100) and Mito fuel flex test (Cat no. 1032870-100) were used following the manufacturer’s instructions to assay fuel utility and mitochondrial metabolism of the Dlat-knockdown 4T1-L or MMTV-L cells.

### Immunohistochemistry (IHC)

Lungs of the mice were analyzed using IHC with anti-Dlat (Proteintech, 13426-1-AP, 1:50), estrogen receptor, progesterone receptor, and HER2 antibodies. Data were captured using Tissue FAXS (TissueGnostics, Vienna, Austria) based on the IHC staining slides. The antibodies used for IHC were listed in Supplementary Table 1.

### Flow cytometry analysis of surface marker expression

THP-1-derived macrophages were incubation with monoclonal antibodies or isotype-matched negative controls for 60 min at 4 °C in the dark. For intracellular staining, cells were fixed using Cytofix and permeabilized with Perm/Wash buffer, according to the manufacturer’s guidelines. Stained cells were then analyzed on a BD Accuri™ flowcytometry (BD Bioscience, San Jose, CA, USA), with a minimum of 10,000 events recorded per sample. The fluorescence-conjugated anti-human antibodies were listed in Supplementary Table 1.

### Statistical analysis

Data analysis was performed using Prism 9.2.0 (GraphPad Software). Unpaired t-tests were employed to compare two groups, while one-way ANOVA was utilized for comparisons involving more than two groups. The results presented in this study were indicative of three independent experiments, demonstrating consistent outcomes. All data are expressed as mean ± standard deviation (SD), and statistical significance was defined as a *p*-value below 0.05.

## Results

### Spatially mapping heterogeneity of metastatic lung nodules

**Fig. 1:**
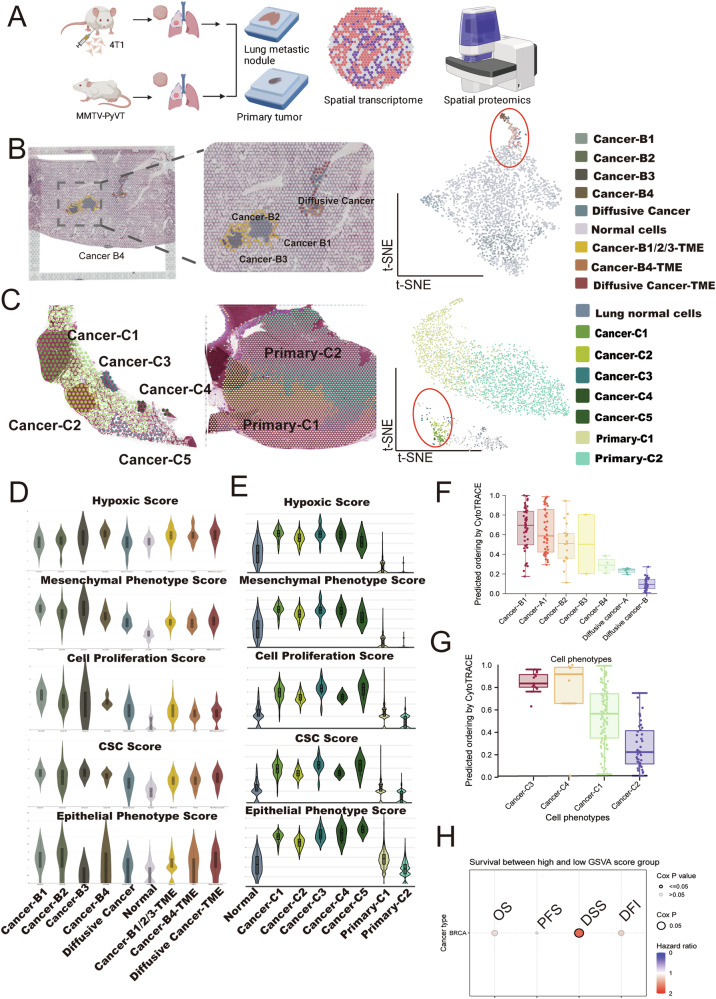
Spatial transcriptomics (ST) of metastatic lung nodules in the 4T1 mouse model. **A** Experimental workflow. Lung sections from two mouse models were utilized to generate spatial data. H&E stain, t-SNE representation, and spatial distribution were identified by clustering the integrated ST dataset across sections from the lungs of the 4T1 model (*n* = 6 for spontaneous metastasis, *n* = 2 for ST) (**B**) and the MMTV-PyVT (**C**) models (*n* = 2 for ST). Malignant status of different tumor nodules in the lung of 4T1 model (**D**) and MMTV-PyVT (**E**) model. CytoTRACE analysis of varying tumor nodules in the lung of the 4T1 model (**F**) and the MMTV-PyVT model (**G**). **H** The survival rate of breast cancer expressing the top 100 genes of aggressive cancer nodules (Cancer-B1). OS, overall survival; PSF, progression-free survival; DSS, disease-specific survival; DFI, disease-free interval.

To investigate the spatial organization of metastatic lung nodules and their microenvironment, we performed spatially resolved transcriptomics on the lungs of mice with two spontaneously metastatic lung nodules in 4T1 (TNBC) and MMTV-PyVT luminal androgen receptor-positive TNBC mouse models [19, 20] (Fig. 1A and Supplementary Fig. 1A). A pathologist examined and annotated the sections based on the morphology of the associated H&E staining and visualized by t-distributed neighbor embedding (t-SNE) plots. Regions were labeled as Cancer-A1 and Diffusive cancer-A (in A section), Cancer-B1 to Cancer-B4 (B section), Diffusive cancer-B (B section), bronchial cells, and lung normal region in 4T1 mouse model (Fig. 1B and Supplementary Fig. 1B, C); Meanwhile, in the MMTV-PyVT mouse model, regions in the lung and mammary gland were labeled as Cancer-C1 to C5, Primary-C1 and Primary-C2, respectively (Fig. 1C). The tumor microenvironments (TMEs) were classified as Cancer-A1-TME and Cancer-B1/2/3-TME, Cancer-B4-TME, and Diffusive cancer-TME in the 4T1 mouse model. TME in the MMTV-PyVT mouse model was assigned as Cancer-C-TME.

To further determine the degree of tumor aggressiveness, we calculated the oncogenic score based on expressed gene sets (hypoxic, mesenchymal, proliferation, cancer stem cells (CSCs), and epithelial phenotype). The results showed that Cancer B1 and Cancer B3 were the most aggressive, exhibiting the highest hypoxic, mesenchymal, and CSC scores, whereas diffusive cancer exhibited the lowest score in the 4T1 mouse model (Fig. 1D, Supplementary Fig. 1D). Similarly, Cancer-C3 in the MMTV-pyVT model was the most aggressive phenotype (Fig. 1E). We applied CytoTRACE to define the degree of tumor aggressiveness based on the number of expressed genes [21]. Consistent with the biological score, Cancer B1 and Cancer C3 had the lowest degree of differentiation in TNBC mouse models, while diffusive cancer cells exhibited high differentiation (Fig. 1F, G). Gene Set Variation Analysis (GSVA) also revealed that the higher score of the top 100 genes of Cancer B1 was associated with poor disease-specific survival (DSS) in breast cancer patients using the TCGA cohort (Fig. 1H).

### The alternative of cell metabolism in lung metastatic tumor nodules

**Fig. 2:**
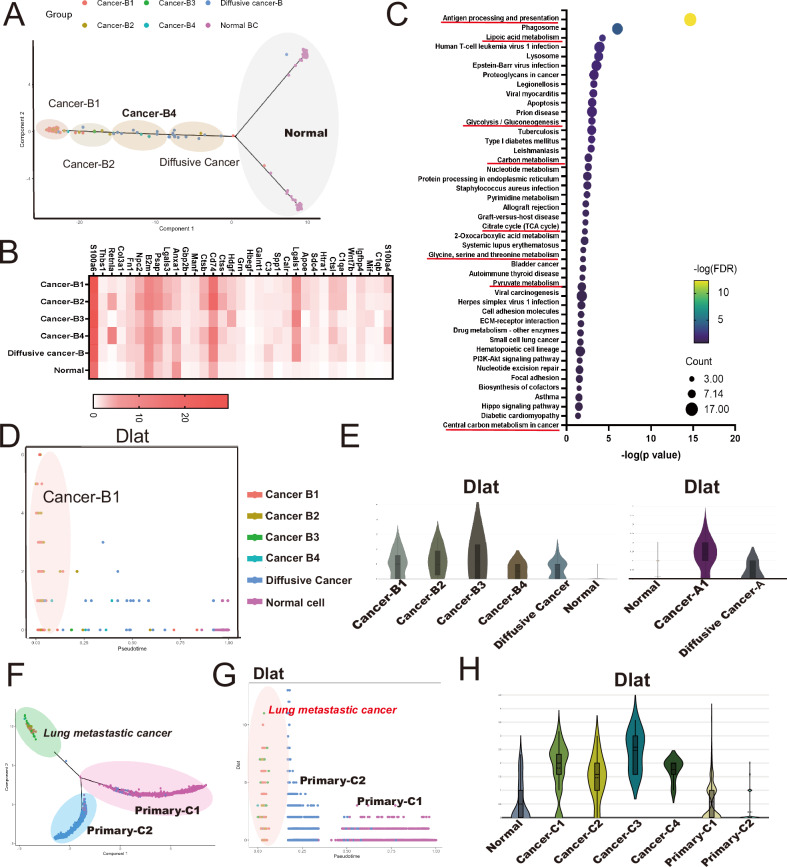
The hub networks contributed to cancers and TMEs. **A** The trajectory analysis of lung metastatic cancer nodules in the 4T1 mouse model. **B** The soluble factors expressed in different tumor nodules of the lung in the 4T1 model. **C** The KEGG pathway of genes contributing to the transition of different tumor nodules in the lung in the 4T1 model. **D** The change of Dlat expression in trajectory analysis. **E** The expression of Dlat in different tumor nodules in the lung of the 4T1 model. **F** The trajectory analysis of primary and lung metastatic tumor nodules in the MMTV-PyVT mouse model. **G** The change of Dlat expression in trajectory analysis and (**H**) The expression of Dlat in different tumor nodules in the primary sites and lung of mice with the MMTV-PyVT mouse model.

To elucidate the spatiotemporally specific transcriptional network and identify key factors in lung metastasis, we conducted Weighted Gene Co-Expression Network Analysis (WGCNA). WGCNA identified two gene modules (Module 1 (M1) and Module 2 (M2)) associated with the degree of tumor aggressiveness in the 4T1 mouse model, as shown in Supplementary Fig. 2A–C. KEGG pathway analysis revealed that M1 was linked to “Pathways in cancer,” “Mitophagy,” “Apoptosis,” and the “Hippo signaling pathway.” It exhibited higher activity in tumor nodules (Supplementary Fig. 2D). The expressions of M2 genes, encoding “Antigen processing and presentation”, “Cell cycle-related pathway,” and several metabolic functions, including “Carbon metabolism”, “TCA cycle”, "Amino acid", and “Fatty acid metabolism”, were widely expressed in lung tumor nodules (Supplementary Fig. 2E). Trajectory analysis further identified 245 genes (*p* < 0.001, *q* < 0.001), including 35 secretory factors, associated with the transition of Cancer-B1 (Fig. 2A, B, and Supplementary Fig. 2F). KEGG pathway analysis indicated the involvement of “Antigen processing and presentation” and several metabolic functions, including “Lipoic acid metabolism”, “Glycolysis/Gluconeogenesis”, “Carbon metabolism”, “TCA cycle”, "Amino acid", and “Fatty acid metabolism” in Cancer-B1 (Fig. 2C). Among them, Dlat, associated with mitochondrial metabolism, was upregulated in all lung cancer nodules in section A and B (Fig. 2D, E). Moreover, trajectory analysis revealed that *Dlat* expression gradually increased according to the aggressiveness of the primary tumor and was higher in all lung metastatic tumor nodules compared with primary mammary tumors in the MMTV-PyVT mouse model (Fig. 2F–H). The data suggested that dysregulation of mitochondrial function induced by Dlat may be associated with lung metastasis in TNBC.

### Upregulation of Dlat was associated with lung metastasis in breast cancer

**Fig. 3:**
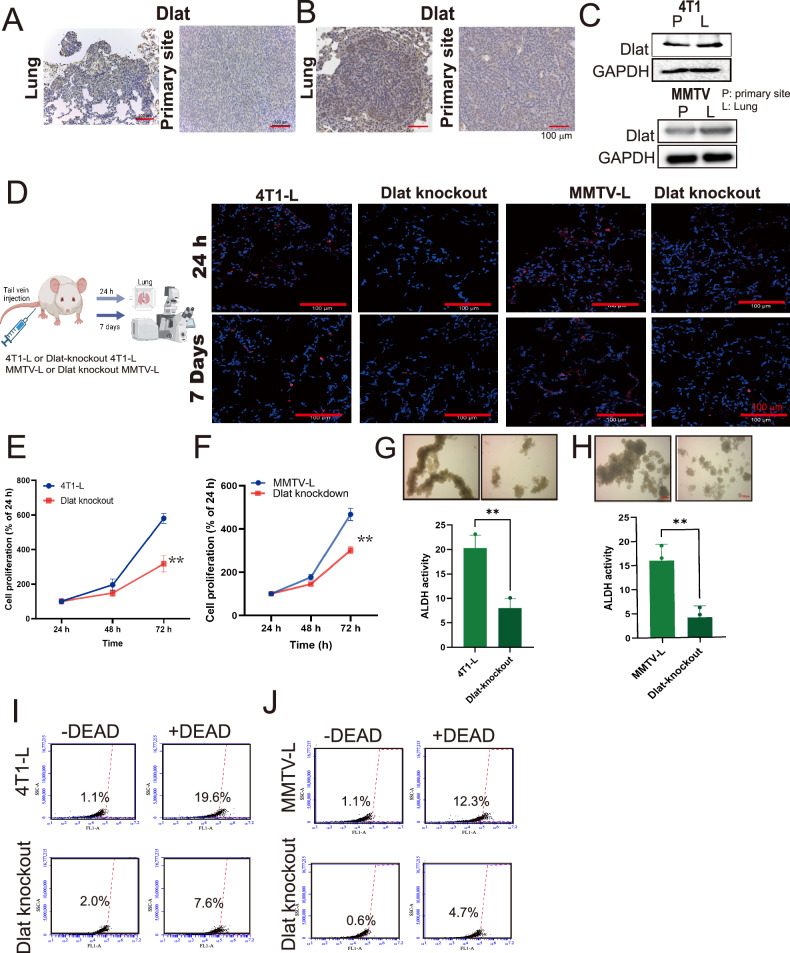
Elevated Dlat associated with lung metastasis of TNBC. The expression of Dlat in the tumor of the primary site and lung nodules in the 4T1 model (*n* = 6) (**A**) and the MMTV-PyVT model (*n* = 6) (**B**). **C** The expression of Dlat in 4T1 cells and MMTV cells. **D** Inhibition of Dlat decreased cell adhesion (24 h) and tumor growth (7 days) in lung of mice. Knockout of Dlat reduced cell proliferation of 4T1-L (**E**) and MMTV-L cells (**F**). Inhibition of Dlat reduced CSC property in 4T1-L (**G**) and MMTV-L cells (**H**) and, as determined by tumor spheroid formation and ALDH activity (**I**, **J**). All results were representative of at least three independent experiments. Graphs shown as mean ± S.D. **p-value < 0.01.

We next evaluated the role of Dlat in lung metastatic and primary tumors in both models. qRT-PCR revealed that the expression of *Dlat* mRNA in the lung metastatic nodules in the 4T1-L and MMTV cells (MMTV-L) isolated from lung tumors compared with corresponding 4T1 and primary MMTV cells (MMTV-P) isolated from primary tumors (Supplementary Fig. 3A, B). Immunohistochemistry (IHC) staining demonstrated increased Dlat proteins observed in the lung tumor nodules of mice with 4T1 breast cancer (Fig. 3A). Compared with primary breast cancer, elevated Dlat was also found in lung metastatic nodules of MMTV-PyVT mice (Fig. 3B). The results were consistent with an increased expression of Dlat protein in the lung metastatic nodules in the 4T1-L and MMTV-L cells (MMTV-L) isolated from lung tumors compared with corresponding 4T1 and primary MMTV cells (MMTV-P) isolated from primary tumors (Fig. 3C).

To validate the role of Dlat in lung metastasis, we established Dlat-knockdown or knockout 4T1-L and MMTV-L cells by either shRNA or CRISPR/Cas9 system (Supplementary Fig. 3D) and then injected them into mice via tail vein. The results indicated that the inhibition of Dlat decreased cell adhesion (24 h post-injection) and cell proliferation (7 days post-injection) in the lungs of mice (Supplementary Fig. 3E and Fig. 3D,). Functional analysis revealed that the inhibition of Dlat reduced cell proliferation and cancer stem cell properties in 4T1-L and MMTV-L cells (Fig. 3E–H, Supplementary Fig. 3F, H). However, the knockdown of Dlat did not affect the ability of cell migration (Supplementary Fig. 3I, J). These results suggested that Dlat is related to cell adhesion and the growth ability of BCs in the pulmonary ecosystem.

### Metabolism alternation in Dlat-upregulated lung metastatic nodules

**Fig. 4:**
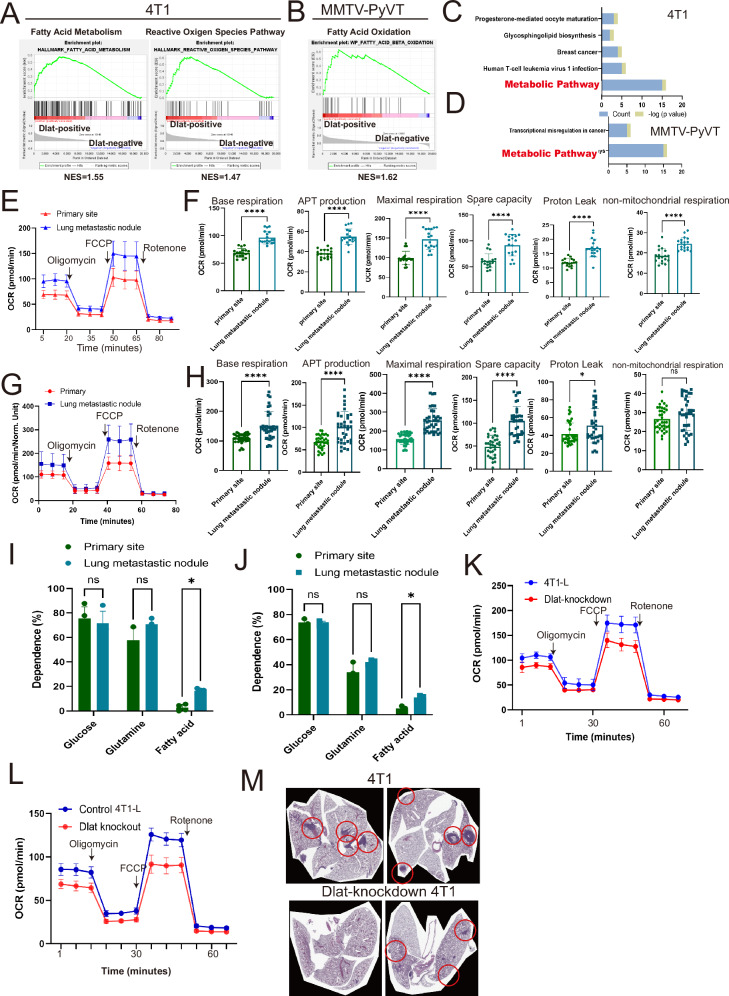
The alternation of lipid metabolism. GSEA analysis of Dlat-positive and negative spots in the lung metastatic nodules in the 4T1 (**A**) and MMTV-PyVT (**B**) mouse model. The KEGG pathway of DEG between Dlat-positive and negative spots in the 4T1 (**C**) and MMTV-PyVT (**D**) mouse models. The oxygen consumption rate (OCR) (**E**), various parameters of respiration (**F**) in 4T1 cells. The OCR (**G**) and various respiration parameters (**H**) in MMTV. Percentage of dependency of mitochondrial respiration to oxidize three main energetic fuels: glucose, glutamine, and fatty acids in 4T1 (**I**) and MMTV (**J**). The OCR of Dlat-knockdown (**K**) and knockout (**L**) 4T1 cells. **M** Knockdown of Dlat reduced lung metastasis in 4T1 cells in vivo (*n* = 6). Graphs shown as means ± SD of experimental triplicates. *p-value < 0.05; **p-value < 0.01; ***p-value < 0.001; ****p-value < 0.0001. DEGs, differentially expressed genes.

Dlat has been indicated to be associated with cell metabolism [22]. Next, we performed Gene Set Enrichment Analysis (GSEA) to explore the biological functions associated with Dlat. Compared with Dlat-negative spots in Cancer-B1 (4T1 model) and Cancer-C3 (MMTV model), Dlat-positive spots in Cancer-B1 in the 4T1 model and Cancer-C3 in the MMTV model exhibited prominent enrichment of signatures related to “Fatty Acid Metabolism” and “ROS Pathway” (Fig. 4A, B, *p* < 0.05). KEGG pathway analysis also showed metabolic pathways related to the differentially expressed genes (DEGs) (Top 100) between Dlat-negative and Dlat-positive cells in both 4T1 and MMTV mice models (Fig. 4C, D, Supplementary Tables 3 and 4). Corresponding to the upregulation of lipid utilization genes, higher oxygen consumption rate (OCR), basal respiration, ATP-linked respiration, maximal respiration, and non-mitochondrial respiration were found in both 4T1-L and MMTV-L cells isolated from the lung, compared with those isolated from the mammary site of the mice, suggesting a greater bioenergetic capacity related to lung metastasis (Fig. 4E–H). However, although there was an upward trend in the extracellular acidification rate (ECAR), it was not statistically significant between 4T1 cells isolated from the lung and those from the primary site (Supplementary Fig. 4). Mitofuel flex assay revealed an increased dependence on fatty acid utilization in 4T1-L and MMTV-L cells isolated from the lung compared with those isolated from the mammary site of the mice (Fig. 4I, J). Lower OCR was observed in 4T1-L cells when Dlat was inhibited by either shRNA transfection or CRISPR/Cas9 knockout (Fig. 4K, L), suggesting that Dlat was associated with regulating metabolism and fuel utility during lung metastasis. More importantly, the knockdown of Dlat decreased lung metastasis in vivo (Fig. 4M), indicating that Dlat-mediated metabolism rewriting was required for lung metastasis in TNBC.

### Increased infiltration of Cd68^+^Cd74^+^ApoE^+^ macrophages of TME surrounding the metastatic lung nodules

**Fig. 5:**
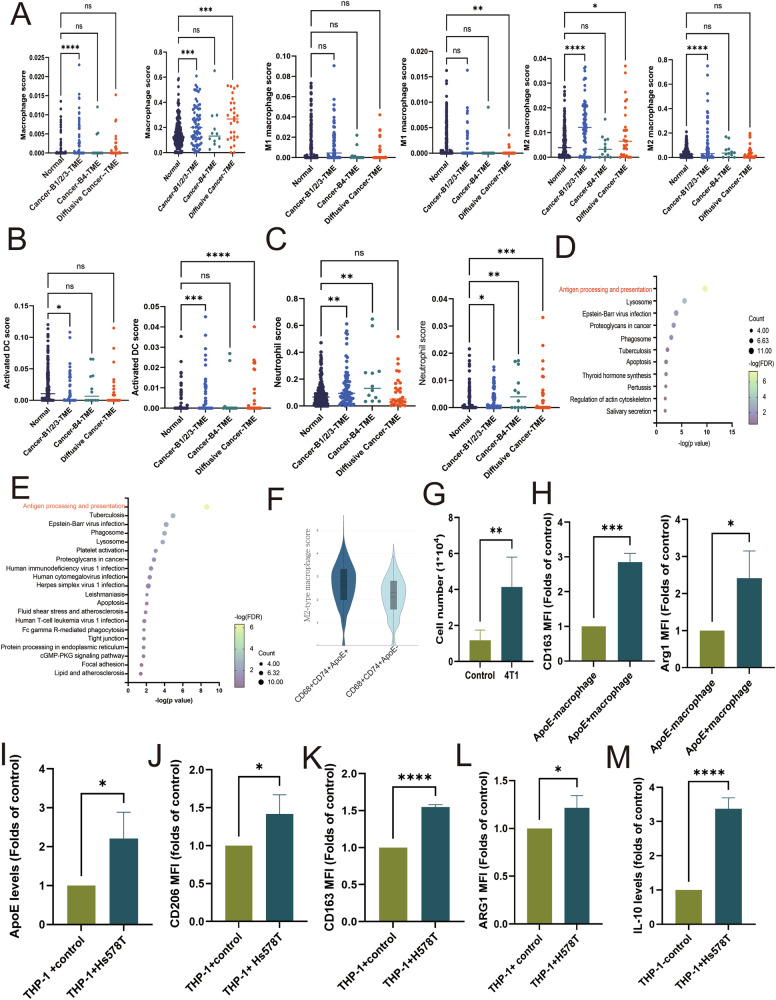
The infiltration of Cd68^+^Cd74^+^ApoE^+^ macrophage in TMEs. The score of macrophages (**A**), dendritic cells (**B**), and Neutrophils (**C**) in TMEs. KEGG pathway of top100 genes of Cancer-B1/2/3-TME (**D**) and Cancer-B4-TME (**E**). **F** M2 macrophage score of Cd68^+^Cd74^+^ApoE^+^ and Cd68^+^Cd74^+^ApoE^-^ spots. **G** The cell number of Cd68^+^Cd74^+^ApoE^+^ macrophages in the lungs of mice (*n* = 10 for control, *n* = 6 for 4T1 model). **H** The expression of M2 markers (*Cd163* and *Arginase I*) in ApoE^+^ and ApoE^-^Cd68^+^Cd74+ cells isolated from the lung of mice with 4T1 (*n* = 6 for 4T1 model). **H** The expression of AopE in THP-1 after Hs578T stimulation. The expression of *ApoE* (**I**), *Cd206* (**J**), *Cd163* (**K**), *Arginase I* (**L**), and *IL-10* (**M**) in macrophage co-cultured with Hs578T cells. All results were representative of at least three independent experiments in vitro. Graphs shown as mean ± S.D. *p-value < 0.05; **p-value < 0.01; ***p-value < 0.001; ****p-value < 0.0001.

Metastatic TME is well known to be a critical regulator of cancer fates [12], and thus, we further assessed the major cell types in TMEs. We sketched the immune profile using the TIMER deconvolution model and the suggested different algorithms (CIBERSORT, XCELL) in TIMER2.0. The results showed that the scores of activated dendritic cells were decreased, whereas M2 macrophages increased in Cancer-B1/2/3-TME and diffusive cancer-TME (Fig. 5A, B, Supplementary Fig. 5A). The scores of M2 macrophages and neutrophils increased, particularly in TME-B1/2/3 (Fig. 5C). However, no differences between all TMEs in T cells, B cells, and cancer-associated fibroblasts (CAFs) were observed (Supplementary Fig. 5B–D). We extracted DEGs from TME-B1/2/3, TME-B4, and TME-Diffusive cancer (*p* < 0.05), and KEGG pathway analysis showed that “Antigen processing and presentation” was involved in all TMEs, suggesting that antigen-presenting cells (APCs) were the major factors for the remodeling of the lung TME for breast cancer spreading (Fig. 5D, E, and Supplementary Fig. 5E). Upregulation of 28 genes was found in all TMEs, compared with normal lung, and macrophage markers such as Cd74, C1qc, and H2-Eb1 increased in all TMEs (Supplementary Fig. 5F). The positive correlation of these three genes with macrophage markers (Cd68), antigen presenting-related factors (H2-Aa, H2-Ab1 H2-Eb1, H2-K, and H2-M3), complement system (C1qc), and lysosomal cysteine proteases (Ctss, Ctsb, Ctsl) as well as phagocytic receptors (Fcgr1 and Fcgr2) were found in all TMEs (Supplementary Fig. 5G). Interestingly, the Cd68 surface marker and lipid-associated marker ApoE also had a strongly positive correlation with Cd74. Most of the spots in TMEs were Cd68^+^Cd74^+^ApoE^+^, which had higher expression of M2 type markers than Cd68^+^Cd74^+^ApoE^-^ spots (Fig. 5F). Macrophages isolated from the lungs of mice with metastatic 4T1 nodules showed an increased number of CD68^+^CD74^+^ApoE^+^ cells, which displayed elevated levels of the M2 markers CD163 and Arginase I (Fig. 5G, H).

To analyze whether breast cancer cells enhanced ApoE expression in macrophages or not and to determine the impact of ApoE on macrophage function, we utilized a THP-1/Hs578T TNBC co-culture model and assessed the effects of ApoE on the macrophage phenotype by blocking. Figure 5I shows that Hs578T breast cancer cells increased the expression of ApoE (Fig. 5I). Furthermore, the expression of M2 markers, including CD206, CD163, arginase I, and IL-10, also increased, while the M1 markers, CD86 and IL-12, decreased (Supplementary Fig. 6A, B). Meanwhile, inhibition of ApoE expression in THP-1 cells blocked the CD86 downregulation and the CD163 upregulation induced by breast cancer cells (Supplementary Fig. 6C–E). We also used the chemical inhibitor to evaluate the metabolism of breast cancer on M2 polarization. The results showed that inhibiting fat utilization can effectively reduce M2 macrophage polarization by Hs578T, which was supported by findings where a fatty acid inhibitor, etomoxir, prevented CD86 downregulation and CD163 upregulation in THP-1 in a co-culture system (Supplementary Fig. 6F, G). These results suggested that macrophages were influenced by breast cancer cells through expressing ApoE and shifted toward the M2 phenotype.

### Upregulated expression of *Lgals1*, *Thbs1*, *S100A* family in macrophages and neutrophils of all TMEs

**Fig. 6:**
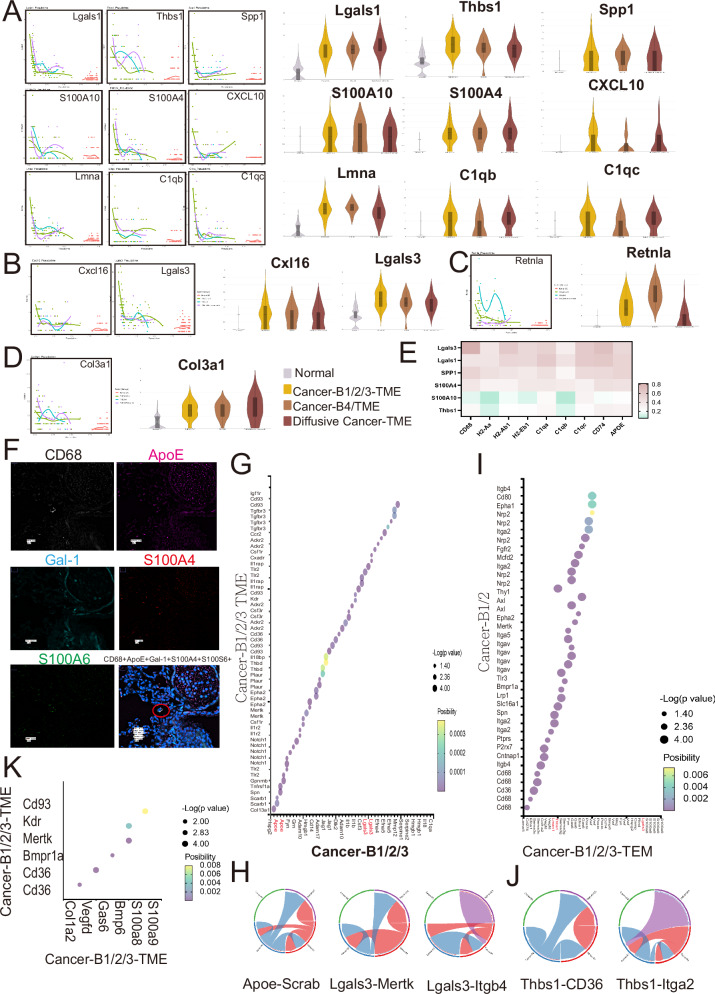
The factors contributed to the remodeling of TMEs. **A** Trajectory analysis of all TMEs. The genes associated with Cancer-B1/2/3-TMEs (**B**), Cancer-B4-TME (**C**), and Diffusive cancer-TMEs (**D**). **E** The correlation of various soluble factors with macrophage markers. **F** The presence of CD68^+^ApoE^+^S100A4^+^S100A6^+^Galectin-1^+^ macrophages in lung TME in vivo (*n* = 6). **G**, **H** The interaction of Cancer-B1/2/3 with their TMEs. **I**, **J** The interactions between Cancer-B1/2/3-TMEs with Cancer-B1/2. **K** The interactions within Cancer-B1/2/3-TMEs.

Next, we applied trajectory analysis to construct the potential transition from normal lung niche to TME. Trajectory analysis revealed that it began as a normal lung and then progressed to a TME diffusive cancer followed by TME-B1/2/3 (Fig. 6A). A total of 117 genes contributed to the transition from normal lung tissue to TME (*p*-value < 0.05; *q*-value < 0.05, supplementary Table 5). Trajectory analysis revealed several soluble factors, including *Lagls1*, *Thbs1*, *Spp1*, *S100* family (*S100A4* and *S100A10*), *Cxcl10*, *Lmna*, and *C1q* (*C1qb*, *C1qc*), were associated with all TMEs. Elevated expressions of all these factors were also found in all TMEs compared with the expressions in normal lungs (Fig. 6A). *Cxcl16* and *Lgals3* were related to Cancer-B1/2/3-TME, while *Retnla* was related to Cancer-B4-TME. *Col3a1* was upregulated in Diffusive cancer-TME (Fig. 6B–D). Interestingly, all of these soluble factors were highly expressed in the lung metastatic nodules of the MMTV-PyVT model (Supplementary Fig. 7). Regression analysis showed that *Lgals3*, *Lgals1*, *Spp1*, and *S100A4*, *S100A6*, had a strong correlation with macrophage markers *Cd68*, *Cd74*, *ApoE*, and lysosome enzymes (Fig. 6E). Multi-immune staining also showed the presence of CD68^+^ApoE^+^Galectin-1^+^S100A4^+^S100A6^+^ macrophages in the metastatic nodule of the lung in 4T1 mouse models (Fig. 6F). The expression of Galectin-1, S100A4, and S100A6 of CD11b^+^CD68^+^ApoE^+^ macrophages was higher compared with the CD11b^+^CD68^+^ApoE^-^ macrophages, as determined by the fluorescence intensity (Supplementary Fig. 8).

To clarify the interaction networks in the TMEs and lung metastatic cancer, we performed ligand-receptor pair analysis on the ST transcriptomic data of all TMs in the 4T1 mouse model. The interactomes revealed a putative interaction between *Lgals3* on neoplastic epithelial cells and its receptor on cell components of Cancer-B1/2/3-TME, respectively (Fig. 6G, H). In addition, the cells of Cancer-B1/2/3 secreted *Thbs1* to interact with Cancer1/2 by *Cd36* and *Itga2* (Fig. 6I, J). *S100A8* and *S100A9/Cd93* were major communication axes within Cancer-B1/2/3-TME (Fig. 6K).

### Exploration of spatial proteasome in TME and lung metastatic BC tumor

**Fig. 7:**
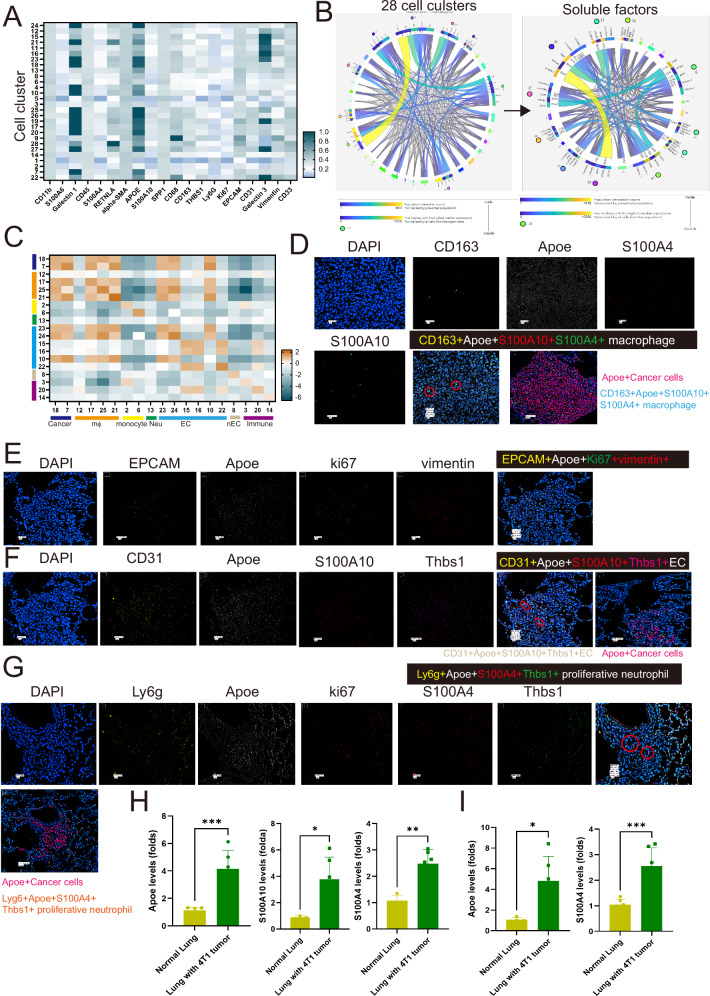
Spatial proteomics of lung metastatic niche. **A** The cell clusters of lung TME were conducted using CODEX staining. **B** The spatial analysis of cell clusters in lung TME. **C** The odd ratio of cell communication between different cell clusters. **D** CD163^+^ApoE^+^ macrophages expressed S100A10 and S100A4 protein. **E** ApoE^+^Ki67^+^ cancer cells expressed higher levels of vimentin. **F** CD31^+^ApoE^+^ endothelial cells expressed S100A10 and Thbs1 protein. **G** The expression of S100A4 and Thbs1 protein in Ly6g^+^ neutrophils. **H** The levels of ApoE, S100A4, and S100A10 in the macrophages isolated from the lung with or without 4T1 tumor nodules (*n* = 6). **I** The levels of ApoE and S100A4 in the neutrophils isolated from the lung with or without 4T1 tumor nodules. Graphs are shown as means ± SD of experimental triplicates. *p-value < 0.05; **p-value < 0.01; ***p-value < 0.001; ****p-value < 0.0001.

Finally, we utilized multiplex immunofluorescence staining, CODEX, to analyze the spatial proteome. Through staining with 21 antibodies determined by ST data, cells were classified into 28 clusters (Fig. 7A). To eliminate bias introduced by the distance between interacting cells, we conducted spatial analysis calculations for cell-cell communication. The results showed that 19 cell clusters, including cancer cells (3 subsets, Ki-67^+-^, Ki67^+^, and Ki67^-^), four groups of macrophages, one group of monocytes, one group of Ly6g^+^ cells (neutrophils), and six groups of endothelial cells (CD31^+^ cells), as well as four immune cell populations, exhibited cell-cell interactions (Fig. 7B and sTable 5). Cluster 25 cells (CD163^+^ApoE^+^ M2 macrophages) and Cluster 18 (cancer cells) showed the most active cellular interactions (Fig. 7C). Cluster 25 (CD163^+^ApoE^+^) macrophages expressed S100A4 and S100A10 (Fig. 7D), while Cluster 18 (ApoE^+^ki67^+^) cancer cells expressed high levels of ApoE, Ki-67, and vimentin (Fig. 7E). Cluster 23 (CD31^+^ApoE^+^ EC cells) also exhibited a significant spatial interaction with Cluster 18 cancer cells by S100A10 and Thbs1 secretion (Fig. 7F). The image data also revealed that Cluster 25/18 or Cluster 23/18 were located in close proximity. Interestingly, we discovered an intriguing subset of proliferative neutrophils (Ly6g^+^ApoE^+^Ki67^+^) that express cell proliferation markers (Ki67) and S100A4 and Thbs1. This specific neutrophil subset exhibited spatial interactions with ApoE^+^ cancer (Cluster 18) (Fig. 7G). Furthermore, upon isolating macrophages and Ly6g^+^ neutrophils from lungs with 4T1 breast cancer metastasis, we found that macrophages expressed higher levels of ApoE, S100A10, and S100A4 than those macrophages isolated from normal mice (Fig. 7H). Additionally, neutrophils isolated from lungs within metastatic 4T1 tumor cells show higher expression of ApoE and S100A4 compared with neutrophils from the lungs of mice without tumors (Fig. 7I).

## Discussion

TNBCs are highly aggressive breast cancers (BCs) with clinical features of high metastatic dissemination, susceptibility to relapse, and poor overall survival [23]. Over the last few years, technological advancements in single-cell profiling and spatial mapping have revolutionized the understanding of tumor heterogeneity and tumor TMEs in primary sites of TNBCs [24, 25]. However, the significance of the spatial organization and tumor heterogeneity of TNBC in the lung has not been investigated. Here, we aimed to characterize both the tumor heterogeneity of lung metastatic nodules and the influence on the tumorigenic TME in the lung. We uncovered insights into the requirement for lipid metabolism rewriting for adhesion and sustained growth during lung metastasis in TNBC. In addition, cell components of the TME, including macrophages, neutrophils, and endothelial cells, contributed to the remodeling of the TME and interactions with cancer cells when they expressed higher levels of lipid metabolism protein, ApoE. Our findings indicate that changes in lipid metabolism play a pivotal role in influencing lung metastasis in both breast cancer cells per se and the cells within the lung metastatic niche.

Currently, the metabolic adaptation of organ-specific metastasis has garnered attention and is considered a crucial factor in enabling tumor cells to successfully establish themselves in a new metastatic microenvironment [26]. In the oxygen-rich environment of the lungs, metastatic cancer cells adapt by shifting their metabolism from glycolysis to oxidative phosphorylation (OXPHOS). Studies have shown that increased lipid utilization plays a pivotal role in driving lung metastasis of breast cancer [27, 28]. A recent study also demonstrated that the lung is a lipid-rich microenvironment, and targeting the process of palmitate inhibits lung metastasis of breast cancer [29]. In contrast, the degradation of lipids by fatty acid amide hydrolase (FAAH) prevents tumor progression and lung metastasis both in vitro and in vivo, and FAAH serves as a biomarker for longer survival in breast cancer patients [30]. Dlat is a component of the pyruvate dehydrogenase (PDH) complex within the TCA cycle and has been reported to promote carcinogenesis by increasing glycolysis [31]. Upregulated Dlat has been reported to be associated with poor clinical outcomes in various cancers, including pancreatic adenocarcinoma, liver cancer, and breast cancer via using bioinformatics analysis [32–34]. In our study, we identified that Dlat and lipid in lung metastatic breast cancer were increased. Notably, increased OCR, ECAR, and lipid dependence were observed in 4T1 and MMTV cells isolated from metastatic lung tumor nodules of mice. Inhibition of Dlat reduced the OCR in TNBC cells, indicating that Dlat upregulation is crucial for metabolic reprogramming during lung metastasis. Dlat knockdown/knockout also diminished the proliferative capacity and cancer stem cell properties of TNBC cells. Also, loss-of-function experiments revealed that Dlat knockdown decreased cell adhesion and inhibited lung tumor growth in vivo. Altogether, these findings provide compelling evidence that alterations in fuel utilization contribute to the pathogenesis of triple-negative breast cancer, particularly lung metastasis, potentially through the modulation of Dlat expression.

Apolipoprotein E (ApoE) has been indicated to be expressed predominantly by tumor-associated macrophages (TAM) or cancer-associated fibroblasts in the tumor in mice or humans [35–37]. Cancer in ApoE knockout (ApoE^−/−^) mice has a lower tumor burden, less fibrosis, reduced innate immune response, and increased adaptive immune cell infiltration while inhibiting ApoE potentiates immune checkpoint inhibitors (ICIs) effectiveness for cancer [36]. Complement C1q (C1q) was also considered to act as a marker of tolerogenic and immunosuppressive macrophage populations in both healthy and tumor tissues. C1q is co-expressed in TAMs with human leukocyte antigen DR (HLA-DR), ApoE, and C1q expression in TAM associates with T cell exhaustion, ICI non-responders, and poor prognosis in patients with malignancy [35, 38, 39]. Huggins et al. reported that lipid-associated TAMs (LAMs, CD68^+^Lagls3^+^Trem2^+^Fabp5^+^), which were observed in the lung of 4T1 nodules, are related to lipid metabolism, extracellular matrix remodeling, and immunosuppression [40]. In our study, we revealed that Cd68^+^ApoE^+^ cells were found in the TMEs of lung metastatic breast tumors were correlated with C1qa/b/c, Cd74, and ApoE expression. Moreover, Cd68^+^Cd74^+^ApoE^+^C1qa^+^ spots exhibited an M2 phenotype and expressed higher levels of galectin-1 and S100A4/6, resulting in immunosuppression in the lung. Our results indicate that Cd68^+^Cd74^+^ApoE^+^ macrophages play a crucial role in breast cancer metastasis to the lungs. However, our findings revealed the upregulation of 28 genes within the lung metastatic TME. Further investigation is required to identify the specific cellular subpopulations in which these genes are expressed.

ApoE is produced and released by various cell types, including astrocytes, microglia, vascular mural cells, hepatocytes, and macrophages [41], and it may play different roles in different cell types. ApoE4 expression in vascular mural cells correlated with the activation of astrocytes, whereas ApoE3 was associated with an angiogenic signature in the cerebrovascular system [41]. ApoE has been reported to play a role in the development of breast cancer [42]. Additionally, in prostate cancer, it has been observed that the secretion of ApoE can regulate the phenotype of neutrophils, promoting an immunosuppressive function [43]. Our results reveal a subset of tumor cells expressing high levels of ApoE, with ApoE-positive endothelial cells (ECs) and ApoE-positive neutrophils positioned close to each other.

Moreover, these ApoE-rich ECs and neutrophils secrete elevated levels of inflammatory substances, including S100A4, S100A10, and Thbs-1. Consequently, these ApoE-rich ECs and neutrophils actively participate in remodeling an immunosuppressive metastatic niche. However, the mechanisms underlying the delivery of ApoE between tumor cells-ECs or tumor cells-neutrophils and the regulation of inflammatory substances in individual cell types by ApoE upregulation, as well as the contribution of all secretory factors in the TME remodeling require further investigation.

In conclusion, this study enhances our understanding of TNBC heterogeneity and maps its microenvironmental landscape using spatial omics approaches. Our findings indicate that the shift from glucose dependence to fatty acids is a critical change for lung metastasis in the TNBC, and the upregulated expression of Dlat plays a vital role in driving this process. Nevertheless, our data depict a detailed landscape of both the most common and the most aggressive tumor microenvironment, untangling the effects of the niche on cancer fate in the lung through various soluble factor-mediated interactions. This study delivers valuable data that serve as an invaluable resource for further research, delineating spatial genomic and proteomic features that can drive the development of therapeutic strategies to improve the clinical outcomes of patients with TNBC.

## Supplementary information

Suppementary file

Western blot

Suppementary Table 3

Suppementary Table 4

Suppementary Table 5

Suppementary Table 6

## Data Availability

The data supporting this study are available upon reasonable request from the corresponding author, Y.L.H.
