# Supplementary material for: Metabolic shifts in lipid utilization and reciprocal interactions within the lung metastatic niche of triple-negative breast cancer revealed by spatial multi-omics
Source: Cell Death Dis. 2024 Dec 18;15(12):899. doi: 10.1038/s41419-024-07205-4 (PMC11655832; doi:10.1038/s41419-024-07205-4)
Supplement: Supplementary file 1 — Suppementary file [file 41419_2024_7205_MOESM1_ESM.pdf]

Supplementary Information:

sFigure 1. Atlas of spatially resolved transcriptomics of metastatic lung nodules in a mouse model. (A) IHC staining of ER, PR and HER-2 in the primary tumor of MMTV-PyVT. (B) H&E and (C) t-SNE representation and spatial distribution as identified by clustering of the integrated ST dataset across sections from lungs of mice. (D) The malignant status of different tumor nodule in the lung. ER, estrogen receptor; PR, progesterone receptor; HER2, human epidermal growth factor receptor 2

sFigure 2. Upregulated Dlat in lung metastatic 4T1 cells. (A) WGCNA of lung tumor nodules and TMEs in the 4T1 model. (B) M1 and (C) M2 module of cancer analyzed by WCGNA. (D) KEGG analysis of M1. (E) The KEGG pathway of M2 module. (F) The genes contributed to the change of different lung metastatic nodule.

sFigure 3. Dlat did not affect the cell migration. The levels of Dlat in breast cancer isolated from primary and lung tumors in the 4T1 model (A) and the MMTV-PyVT model (B). (C) The knockout of Dlat in 4T1 cells. (E) The knockout of Dlat in 4T1-L and MMTV-L cells. (F) The cell proliferation of 4T1-L and Dlat-knokdown 4T1-L cells. (F) The cancer stem cell property of 4T1 and Dlat-knockdown 4T1-L cells, as determined by tumor spheroid (G) and ALDH activity (H). The effect of Dlat in cell migration, as determined by wound healing analysis (I) and Transwell migration analysis (J). ns. no significant difference. All results were representative of at least three independent experiments *in vitro*. Graphs shown as mean ± S.D. **, p value < 0.01; ***, p value < 0.001

sFigure 4. The ECARD of 4T1 and 4T1-L cell.

sFigure 5. The immune profile of all TMEs. (A) The distribution of TMEs. (B) The T cell score of TMEs. (C) The B cell score of TMEs. (D) The cancer-associated fibroblast (CAF) score of TMEs. (E) The KEGG pathway of top 100 genes of Cancer-B4 TME. (F) The common genes in all TMEs (G) The correlation of various genes. ns. No significant difference.

sFigure 6. The role of ApoE in macrophages. The level of CD168 (A) and IL-12 in THP-1 co-cultured with Hs578T. Inhibition of ApoE by siRNA transfection (C) prevented the polarization of macrophages by Hs578T, supported by CD86 downregulation (D) and, CD163 upregulation (E). The fatty acid oxidation (FAO) inhibitor, ETOMXIR, decreased the effect of TNBC Hs578T breast cell in the downregulation of CD86 (F) and Upregulation of CD163 in THP-1 (G). Hs578T cells

were treated with etomxir (4 μM) for 24 hours. After washing, cells were co-cultured with THP-1-derived macrophages for 24 hours. The expression levels of CD83 and CD86 were assessed by a flow-cytometry. All results were representative of at least three independent experiments *in vitro*. Graphs shown as mean ± S.D. **, p value < 0.01; ***, p value < 0.001

sFigure 7. The expression of various factors in the TME of lung tumor nodules of MMTV-PyVT mice.

sFigure 8. Higher levels of Galectin-1, S100A4 and S100A6 in $CD68^{+}ApoE^{+}$ macrophages.

sTable 1. The antibody list used in this study

sTable 2. The primer list

sTable 3. The DEGs of Dlat-positive and negative spots in the 4T1 model

sTable 4. The DEGs of Dlat-positive and negative spots in the MMTV-PyVT model

sTable 5**.** The genes contributed the change of TMEs.

sTable 6. Cell clusters

sTable 1. The list of antibodies used in this study

Antibodies used in Western blot			
**Antibody**	**Company**	**Catalog**	**Dilution**
Dlat	Proteintech	13426-1-AP	1:2000

Antibodies used in IHC			
**Antibody**	**Company**	**Catalog**	**Dilution**
Dlat	Proteintech	13426-1-AP	1:100
ER	Proteintech	20698-1-AP	1:50
PR	Proteintech	25871-1-AP	1:50
Her2	Proteintech	18299-1-AP	1:50

Antibody list used in CODEX system						
**Antibody**	**Company**	**Catalog**	**Species**	**Barcode**	**Conjugation Color**	**Dilution**
CD31	AKOYA	232163	Mouse	BX002	Atto 550	1:200
Galectin 3	abcam	ab2785	Mouse, Human	BX003	Cy5	1:250
Vimentin	abcam	ab193555	Mouse, Human	BX005	Atto 550	1:500
CD45	AKOYA	232126	Mouse	BX007	AF488, AF750	1:200
EPCAM	abcam	ab232539	Mouse, Human	BX010	AF488	1:100
SPP1	abcam	ab283669	Mouse, Human	BX022	AF647	1:2000
Ly6G	AKOYA	232163	Mouse	BX024	AF647	1:200
S100A6	abcam	ab181975	Mouse, Human	BX026	Atto 550	1:100
CD11c	AKOYA	232169	Mouse	BX030	Cy5	1:200
CD163	abcam	ab182422	Mouse, Human	BX031	AF647	1:300
RELM alpha (Retnla)	abcam	AB39626	Mouse	BX036	AF647	1:200
ApoE	Cell Signaling	20769	Mouse	BX040	Atto 550	1:200
Ki67	AKOYA	232163	Mouse	BX047	Atto 550	1:200
Thrombosp	thermofish	MA5-	Mouse,	BX049	Atto 550	1:50

ondin 1(THBS1)	er	13398	Human			
CD68	abcam	ab125212	Mouse	BX052	Atto 550	1:100
Galectin 1	abcam	ab240111	Mouse, Human	BX054	AF647	1:16000
S100A10	abcam	ab232524	Mouse, Human	BX055	AF647	1:100
S100A4	abcam	ab220213	Mouse, Human	BX041	Atto 550	1:2000
alpha-SMA	abcam	ab240654	Mouse, Human	BX013	AF488, AF750	1:50
CD11b	AKOYA	232164	Mouse	BX025	AF488, AF750	1:200
CD33	thermofisher	PA5-120758	Mouse, Human	BX043	Atto 550	1:200

Antibodies used in Flow cytometry

**Antibody**	**Cat No.**	**Volume**
BD Pharmingen™		
BD Cytofix/Cytoperm™ Fixation/Permeabilization Kit	554714	
APC Mouse Anti-Human CD83	551073	20ul
APC Mouse IgG1, κ Isotype Control RUO	555751	20ul
FITC Mouse Anti-Human CD206	551135	20 µl
FITC Mouse IgG1, κ Isotype Control RUO	555748	20 µl
Mouse Anti-Human CD163	556018	20 µl
PE Mouse IgG1, κ Isotype Control RUO	555749	20 µl
PerCP-Cy™5.5 Mouse Anti-Human CD86	561129	5 µl
PerCP-Cy™5.5 Rat IgG1, λ Isotype Control	551072	5 µl
PE Mouse Anti-Human CD86	555658	20 µl
abcam		
Anti-Apolipoprotein E antibody	ab183597	1:70

[EPR19392]		
APC Rabbit IgG, monoclonal [EPR25A] - Isotype Control	ab232814	1:70
Goat Anti-Rabbit IgG H&L (APC) preadsorbed	ab130805	1:500<br>0.1 µg/10^6 cells
Invitrogen		
Arginase 1 Monoclonal Antibody (A1exF5), PE-Cyanine7, eBioscience™	25-3697-82	0.5 µg
Rat IgG2a kappa Isotype Control (eBR2a), PE-Cyanine7, eBioscience™	25-4321-81	0.5 µg

sTable 2. The primers used in this study

Dlat_Mus_F1	AGGTGCTGTTGGTACGGAAG
Dlat_Mus_R1	GCAACACTGACGTCAACCAC
ApoE_F1	CGCTTTTGGGATTACCTGCG
ApoE_R1	GGGGTCAGTTGTTCCTCCAG
GAPDH-F	AACTTTGGCATTGTGGAAGG
GAPDH-reverse	ACACATTGGGGGTAGGAACA

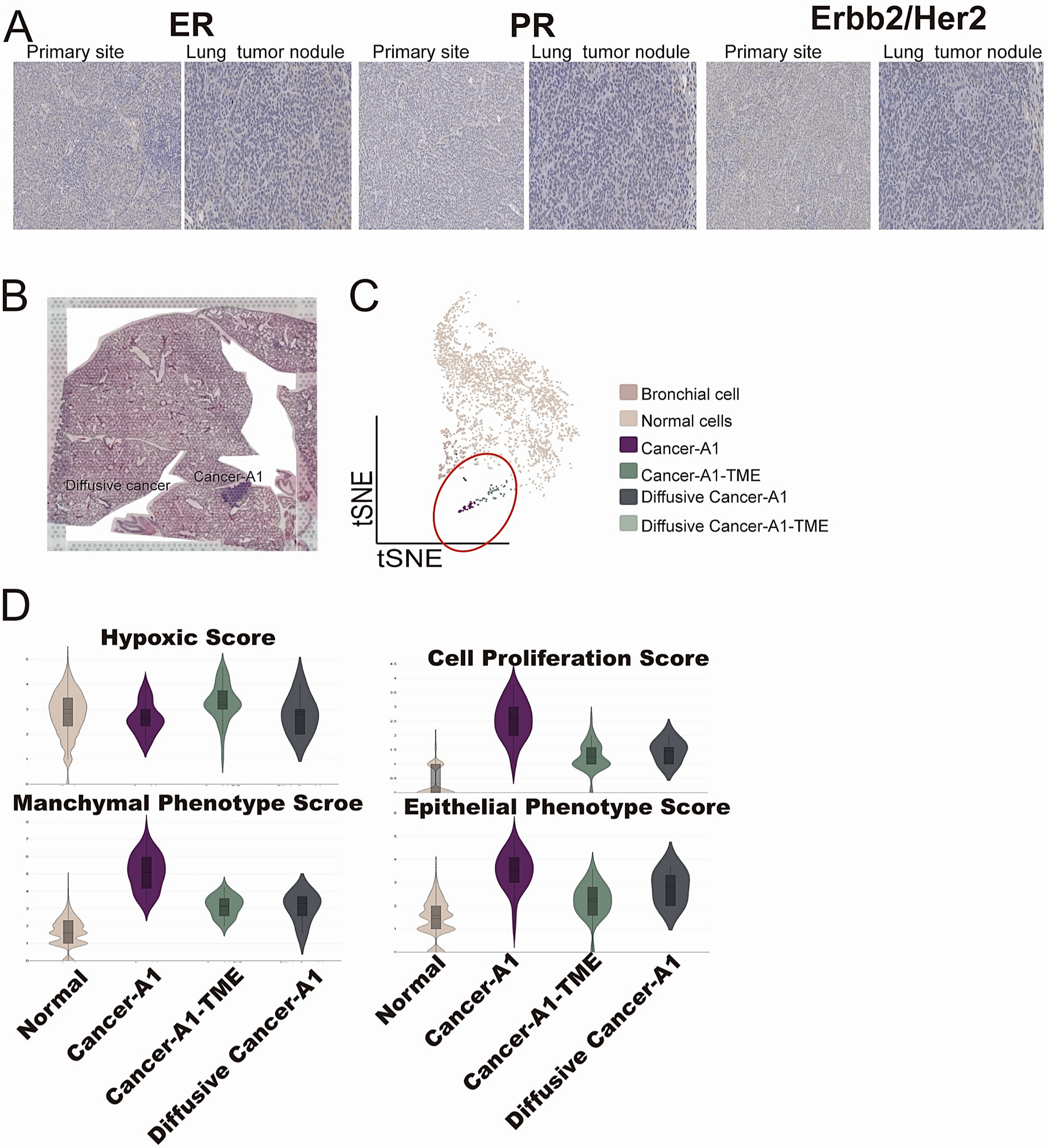
A
ER
PR
Erbb2/Her2
Primary site
Lung tumor nodule
B
Diffusive cancer
Cancer-A1
C
tSNE
tSNE
Bronchial cell
Normal cells
Cancer-A1
Cancer-A1-TME
Diffusive Cancer-A1
Diffusive Cancer-A1-TME
D
Hypoxic Score
Cell Proliferation Score
Manchymal Phenotype Scroe
Epithelial Phenotype Score
Normal
Cancer-A1
Cancer-A1-TME
Diffusive Cancer-A1

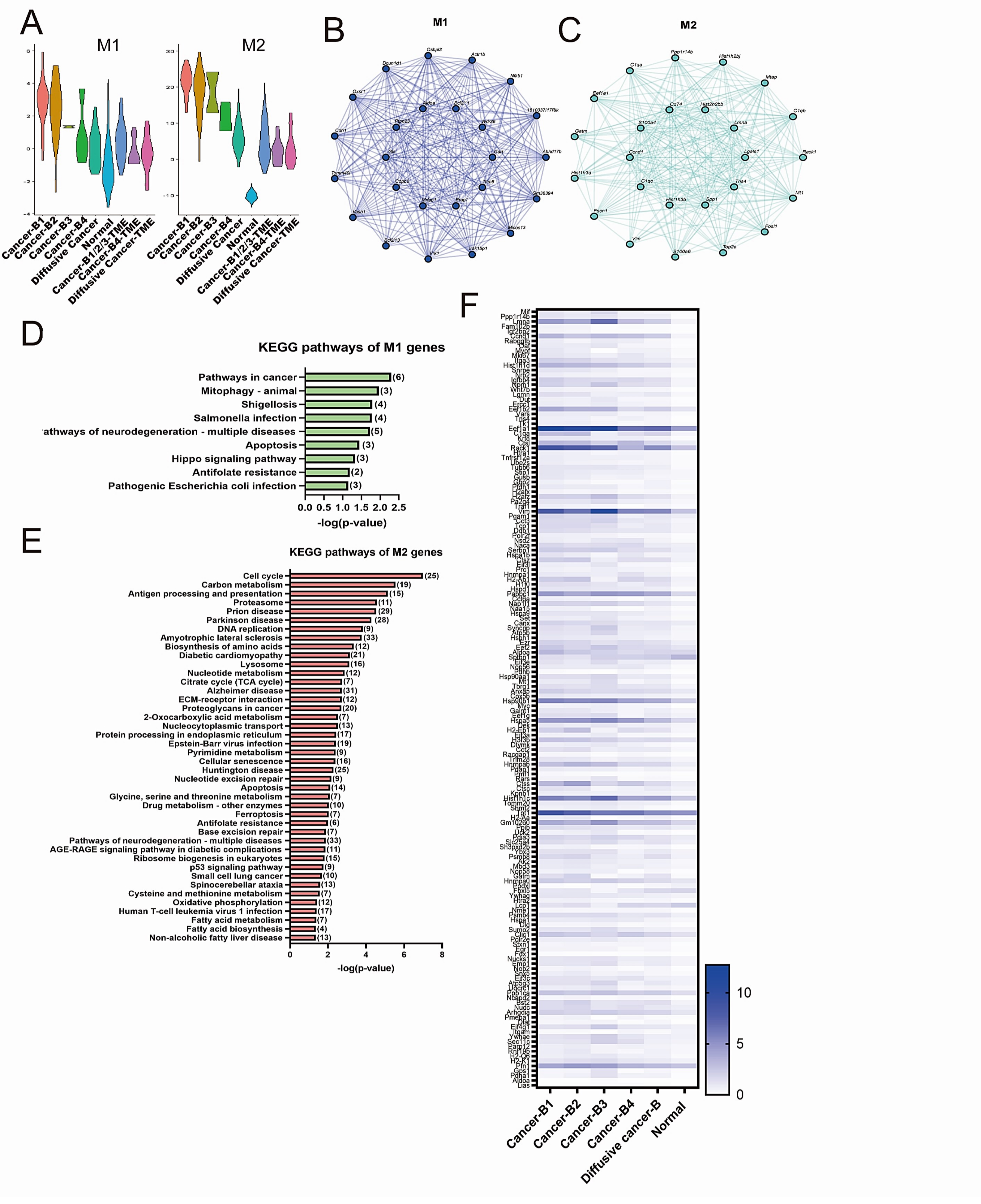
A
M1
M2
Cancer-B1
Cancer-B2
Cancer-B3
Cancer-B4
Diffusive Cancer
Normal
Cancer-B1/2/3-TME
Cancer-B4-TME
Diffusive Cancer-TME
B
M1
C
M2
D
KEGG pathways of M1 genes
Pathways in cancer (6)
Mitophagy - animal (3)
Shigellosis (4)
Salmonella infection (4)
athways of neurodegeneration - multiple diseases (5)
Apoptosis (3)
Hippo signaling pathway (3)
Antifolate resistance (2)
Pathogenic Escherichia coli infection (3)
-log(p-value)
E
KEGG pathways of M2 genes
Cell cycle (25)
Carbon metabolism (19)
Antigen processing and presentation (15)
Proteasome (11)
Prion disease (29)
Parkinson disease (28)
DNA replication (9)
Amyotrophic lateral sclerosis (33)
Biosynthesis of amino acids (12)
Diabetic cardiomyopathy (21)
Lysosome (16)
Nucleotide metabolism (12)
Citrate cycle (TCA cycle) (7)
Alzheimer disease (31)
ECM-receptor interaction (12)
Proteoglycans in cancer (20)
2-Oxocarboxylic acid metabolism (7)
Nucleocytoplasmic transport (13)
Protein processing in endoplasmic reticulum (17)
Epstein-Barr virus infection (19)
Pyrimidine metabolism (9)
Cellular senescence (16)
Huntington disease (25)
Nucleotide excision repair (9)
Apoptosis (14)
Glycine, serine and threonine metabolism (7)
Drug metabolism - other enzymes (10)
Ferroptosis (7)
Antifolate resistance (6)
Base excision repair (7)
Pathways of neurodegeneration - multiple diseases (33)
AGE-RAGE signaling pathway in diabetic complications (11)
Ribosome biogenesis in eukaryotes (15)
p53 signaling pathway (9)
Small cell lung cancer (10)
Spinocerebellar ataxia (13)
Cysteine and methionine metabolism (7)
Oxidative phosphorylation (12)
Human T-cell leukemia virus 1 infection (17)
Fatty acid metabolism (7)
Fatty acid biosynthesis (4)
Non-alcoholic fatty liver disease (13)
-log(p-value)
F
Cancer-B1
Cancer-B2
Cancer-B3
Cancer-B4
Diffusive cancer-B
Normal

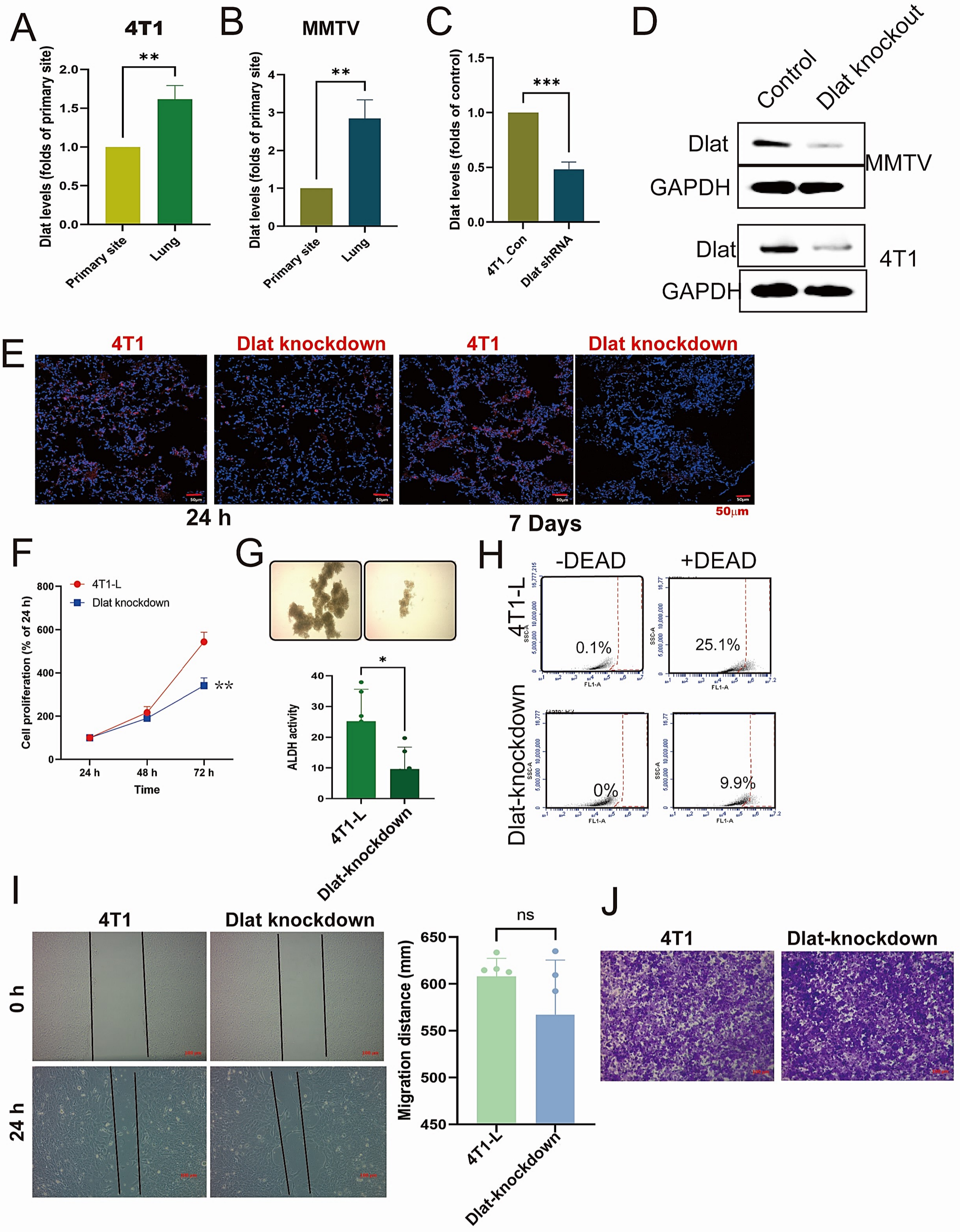
A
4T1
Dlat levels (folds of primary site)
**
Primary site
Lung
B
MMTV
Dlat levels (folds of primary site)
**
Primary site
Lung
C
Dlat levels (folds of control)
***
4T1_Con
Dlat shRNA
D
Control
Dlat knockout
Dlat
GAPDH
MMTV
Dlat
GAPDH
4T1
E
4T1
Dlat knockdown
4T1
Dlat knockdown
24 h
7 Days
50µm
F
4T1-L
Dlat knockdown
Cell proliferation (% of 24 h)
**
24 h
48 h
72 h
Time
G
ALDH activity
*
4T1-L
Dlat-knockdown
H
-DEAD
+DEAD
4T1-L
Dlat-knockdown
0.1%
25.1%
0%
9.9%
I
4T1
Dlat knockdown
0 h
24 h
Migration distance (mm)
ns
4T1-L
Dlat-knockdown
J
4T1
Dlat-knockdown

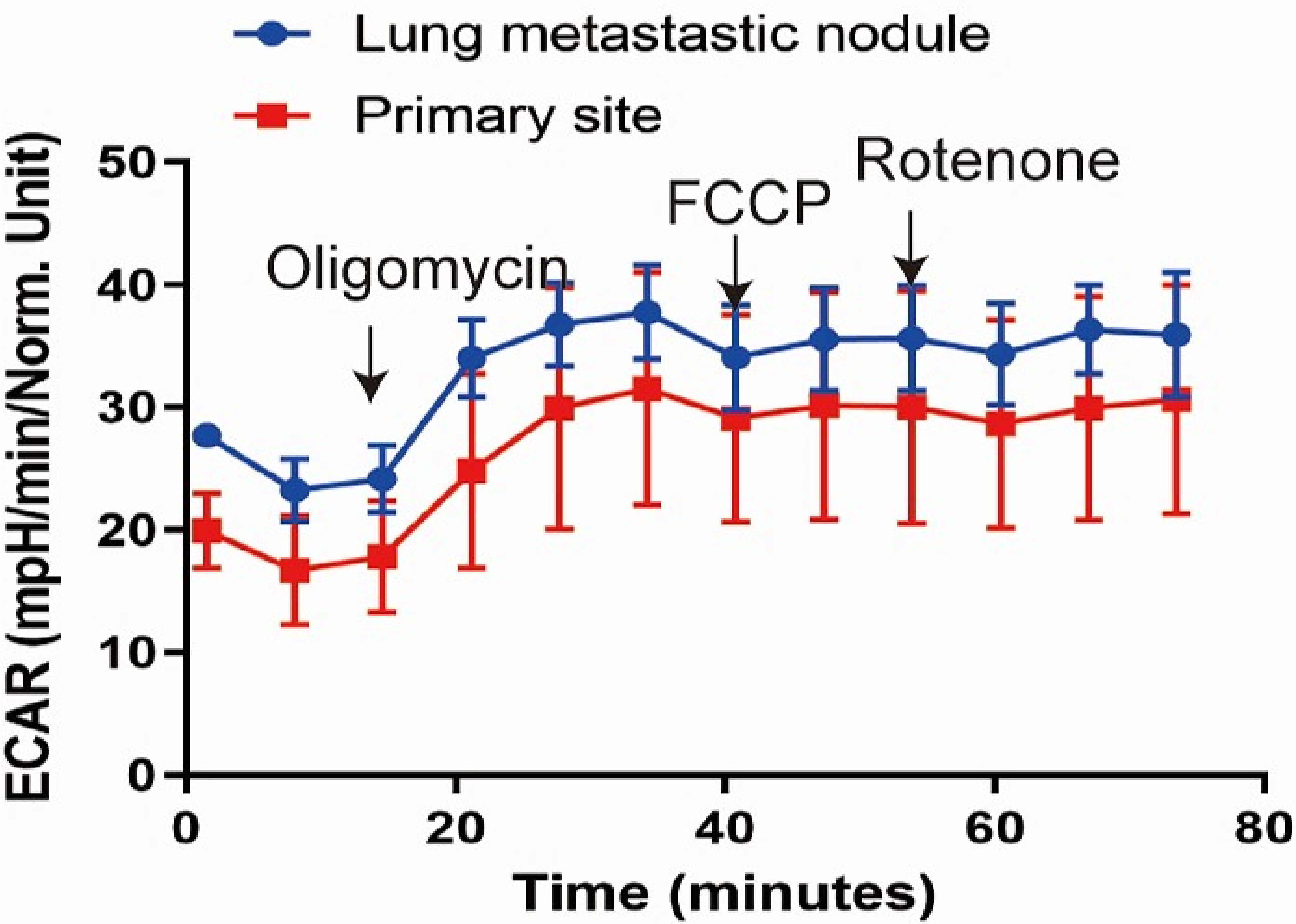

Lung metastastic nodule
Primary site
ECAR (mpH/min/Norm. Unit)
50
40
30
20
10
0
Oligomycin
FCCP
Rotenone
0
20
40
60
80
Time (minutes)

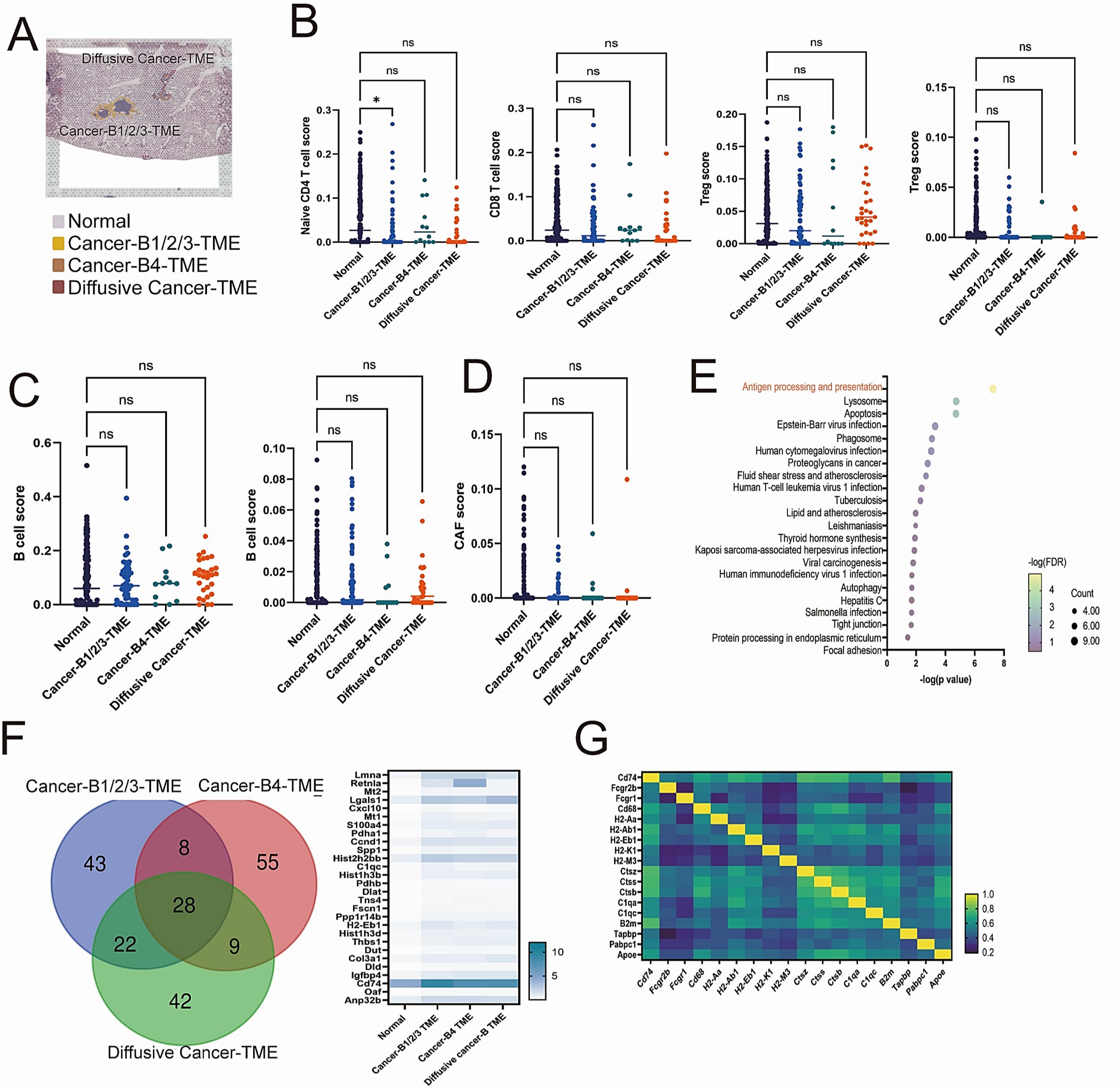
A
Diffusive Cancer-TME
Cancer-B1/2/3-TME
Normal
Cancer-B1/2/3-TME
Cancer-B4-TME
Diffusive Cancer-TME
B
Naive CD4 T cell score
CD8 T cell score
Treg score
C
B cell score
D
CAF score
E
Antigen processing and presentation
Lysosome
Apoptosis
Epstein-Barr virus infection
Phagosome
Human cytomegalovirus infection
Proteoglycans in cancer
Fluid shear stress and atherosclerosis
Human T-cell leukemia virus 1 infection
Tuberculosis
Lipid and atherosclerosis
Leishmaniasis
Thyroid hormone synthesis
Kaposi sarcoma-associated herpesvirus infection
Viral carcinogenesis
Human immunodeficiency virus 1 infection
Autophagy
Hepatitis C
Salmonella infection
Tight junction
Protein processing in endoplasmic reticulum
Focal adhesion
-log(p value)
-log(FDR)
Count
F
Cancer-B1/2/3-TME
Cancer-B4-TME
Diffusive Cancer-TME
43
8
55
28
22
9
42
G

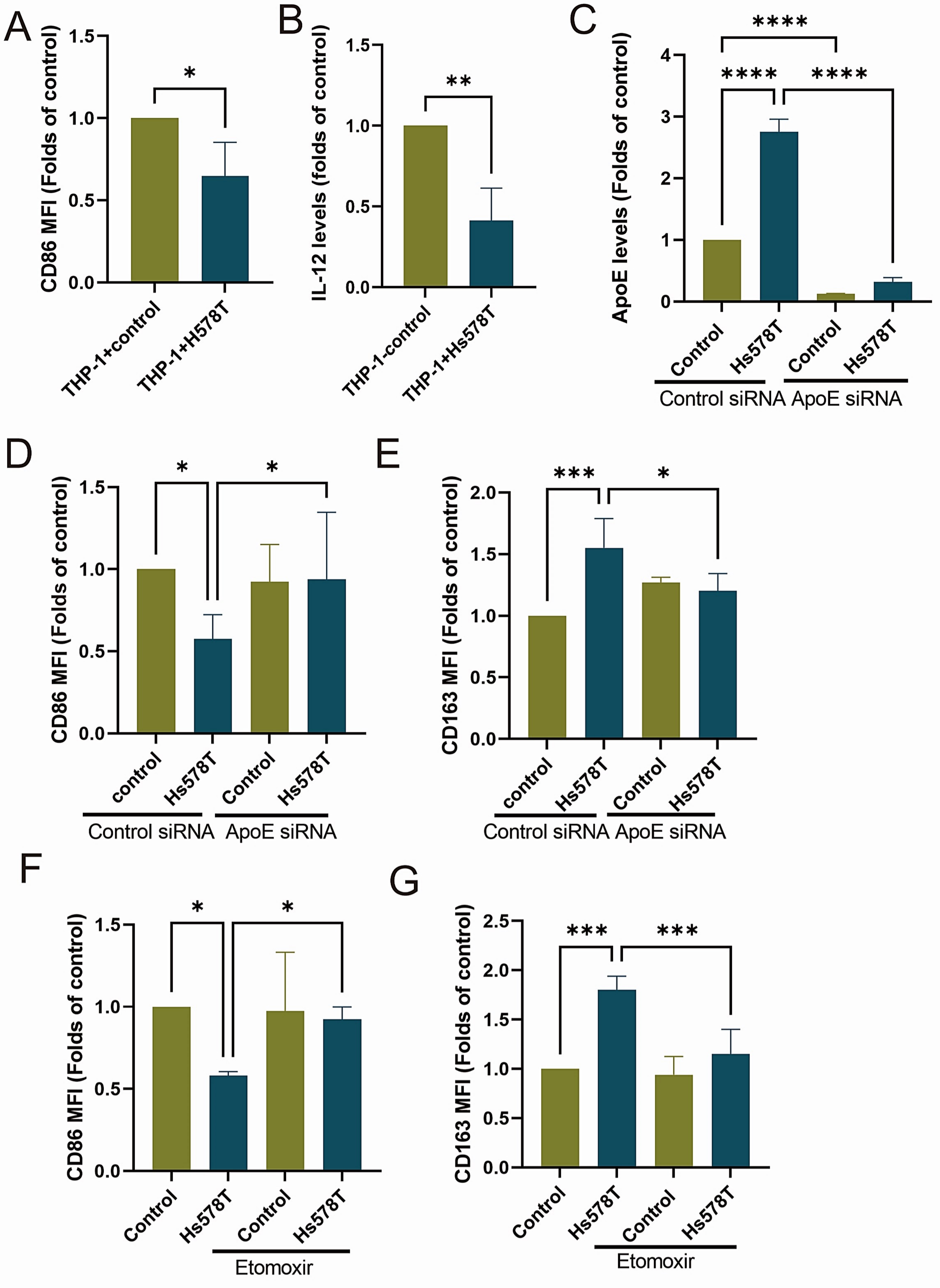
A
CD86 MFI (Folds of control)
1.5
1.0
0.5
0.0
*
THP-1+control
THP-1+H578T
B
IL-12 levels (folds of control)
1.5
1.0
0.5
0.0
**
THP-1-control
THP-1+Hs578T
C
ApoE levels (Folds of control)
4
3
2
1
0
****
****
****
Control
Hs578T
Control
Hs578T
Control siRNA ApoE siRNA
D
CD86 MFI (Folds of control)
1.5
1.0
0.5
0.0
*
*
control
Hs578T
Control
Hs578T
Control siRNA ApoE siRNA
E
CD163 MFI (Folds of control)
2.0
1.5
1.0
0.5
0.0
***
*
control
Hs578T
Control
Hs578T
Control siRNA ApoE siRNA
F
CD86 MFI (Folds of control)
1.5
1.0
0.5
0.0
*
*
Control
Hs578T
Control
Hs578T
Etomoxir
G
CD163 MFI (Folds of control)
2.5
2.0
1.5
1.0
0.5
0.0
***
***
Control
Hs578T
Control
Hs578T
Etomoxir

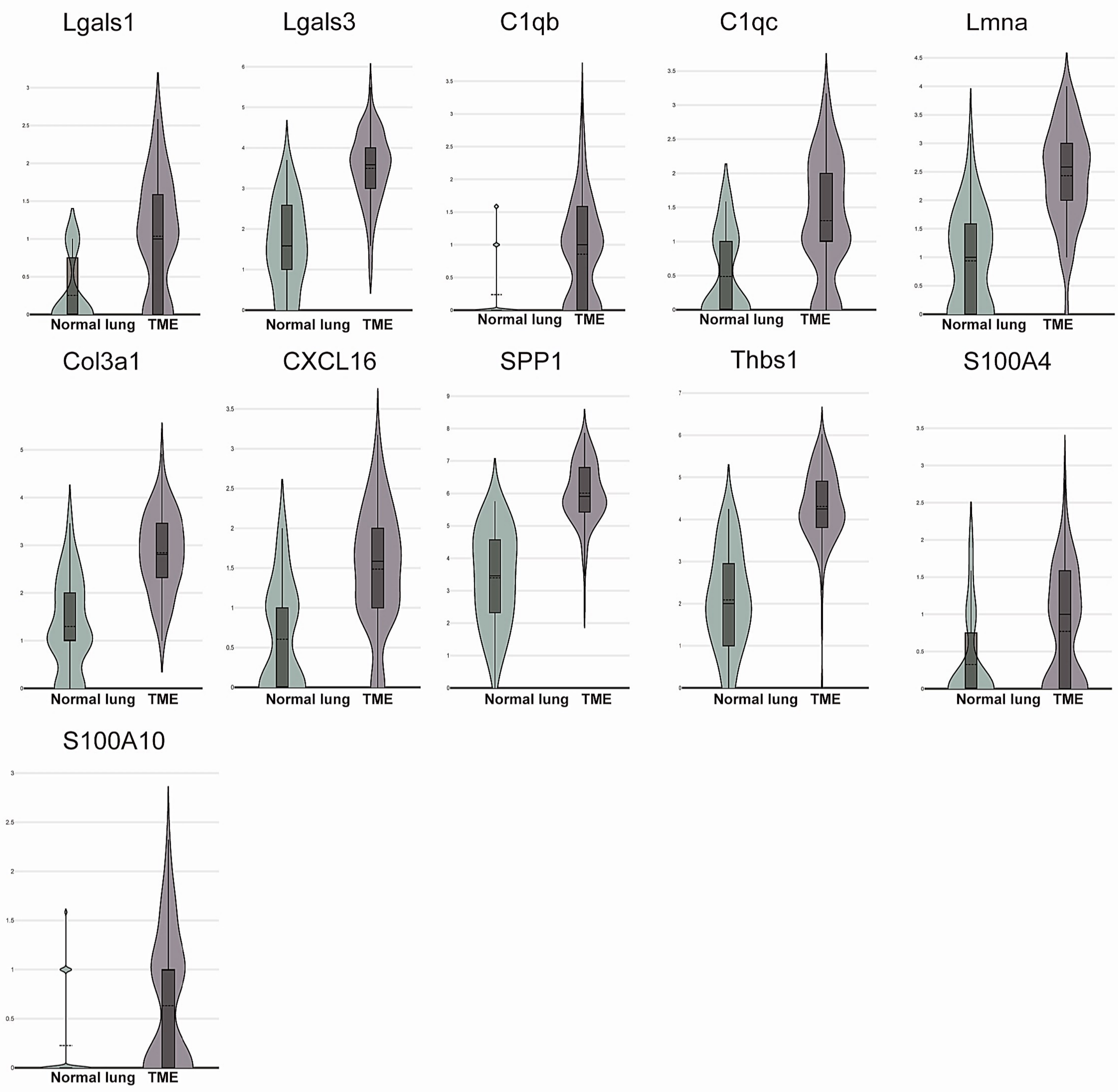

Lgals1
Lgals3
C1qb
C1qc
Lmna
Col3a1
CXCL16
SPP1
Thbs1
S100A4
S100A10
Normal lung
TME

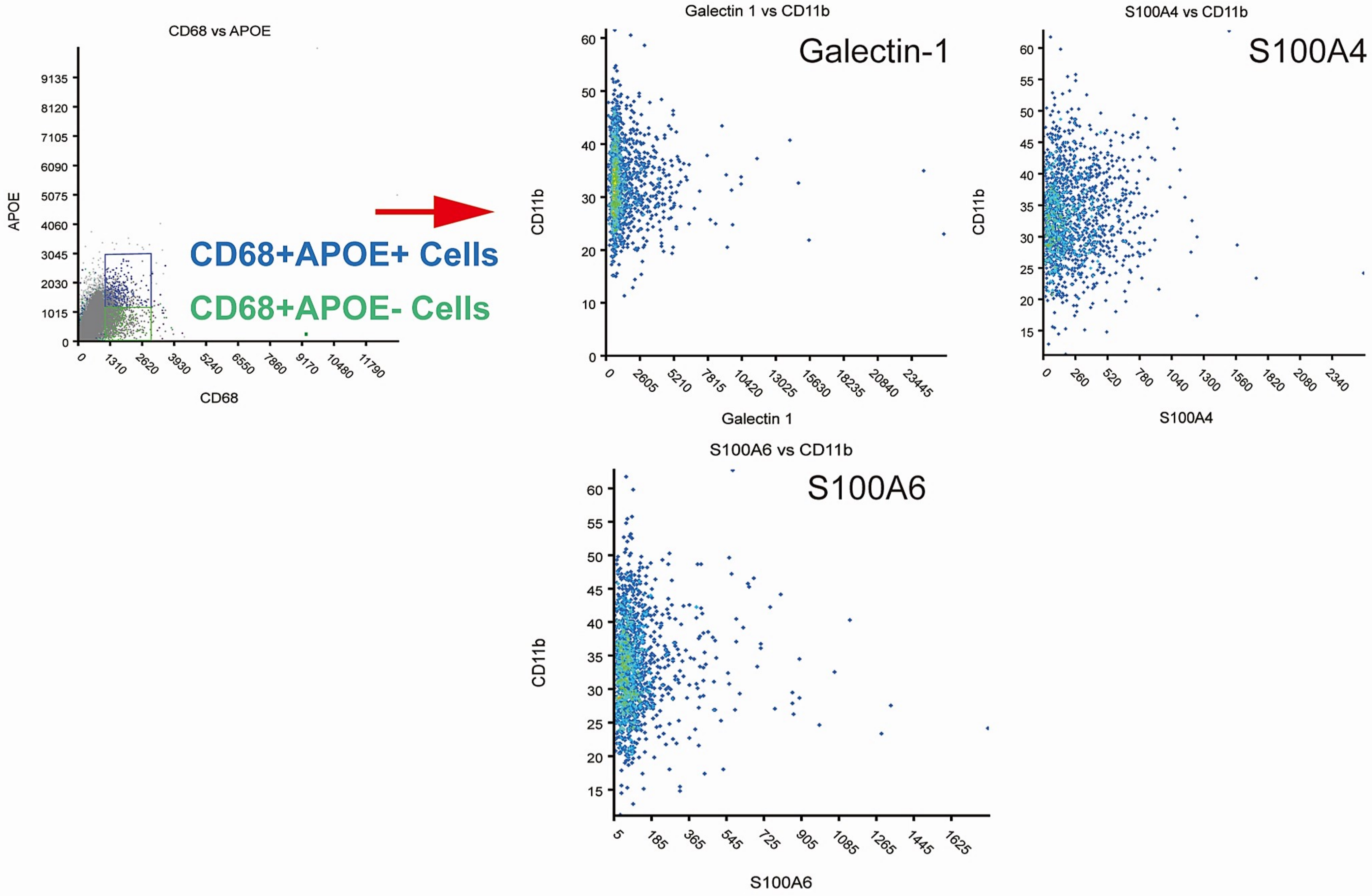
CD68 vs APOE
APOE
CD68
9135
8120
7105
6090
5075
4060
3045
2030
1015
0
1310
2620
3930
5240
6550
7860
9170
10480
11790
CD68+APOE+ Cells
CD68+APOE- Cells
Galectin 1 vs CD11b
Galectin-1
CD11b
Galectin 1
60
50
40
30
20
10
2605
5210
7815
10420
13025
15630
18235
20840
23445
S100A4 vs CD11b
S100A4
55
45
35
25
15
260
520
780
1040
1300
1560
1820
2080
2340
S100A6 vs CD11b
S100A6
5
185
365
545
725
905
1085
1265
1445
1625
