# Supplementary figures and images for: Metabolic shifts in lipid utilization and reciprocal interactions within the lung metastatic niche of triple-negative breast cancer revealed by spatial multi-omics
Source: Cell Death Dis. 2024 Dec 18;15(12):899. doi: 10.1038/s41419-024-07205-4 (PMC11655832; doi:10.1038/s41419-024-07205-4)

Figure 3C

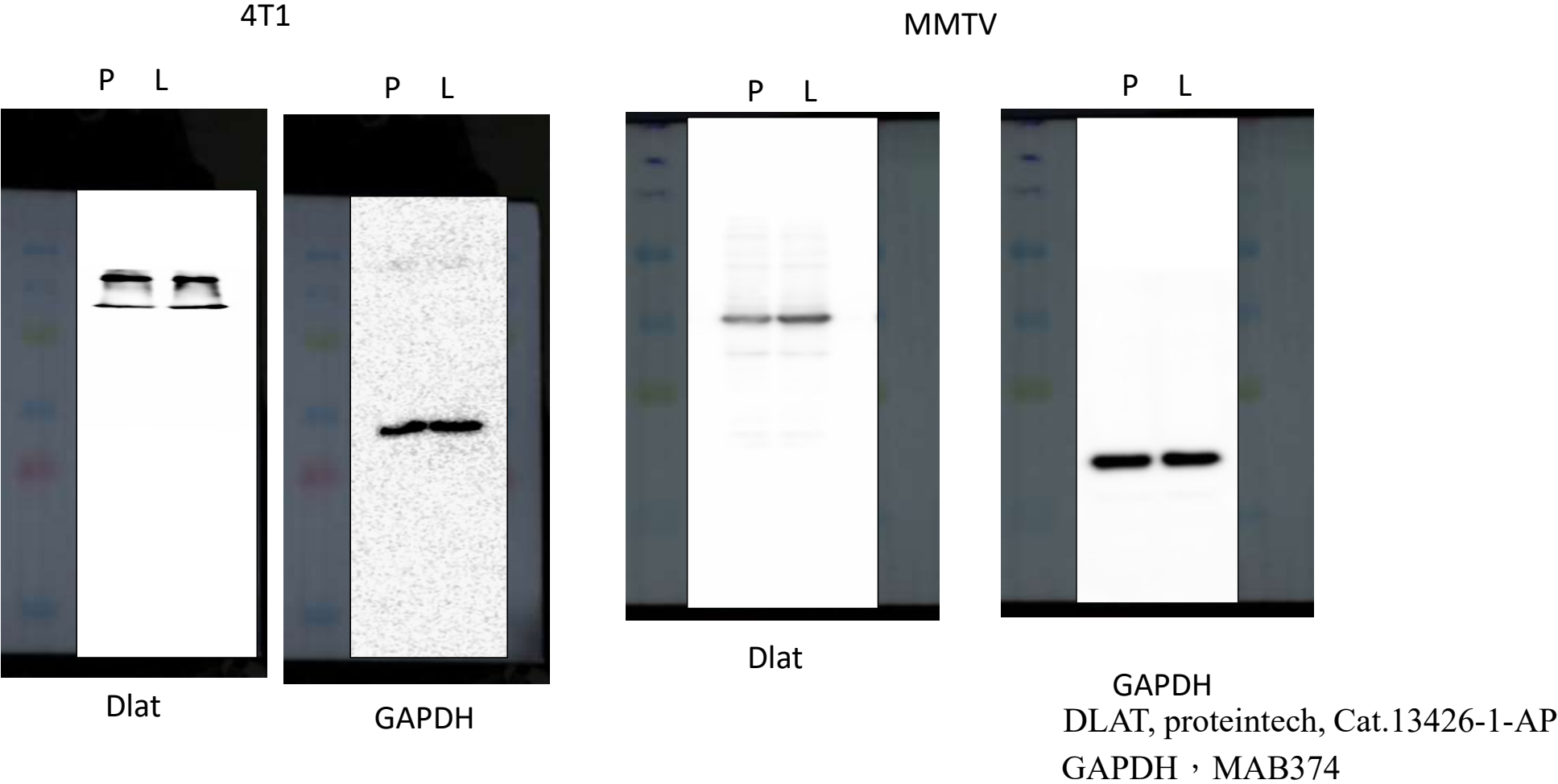

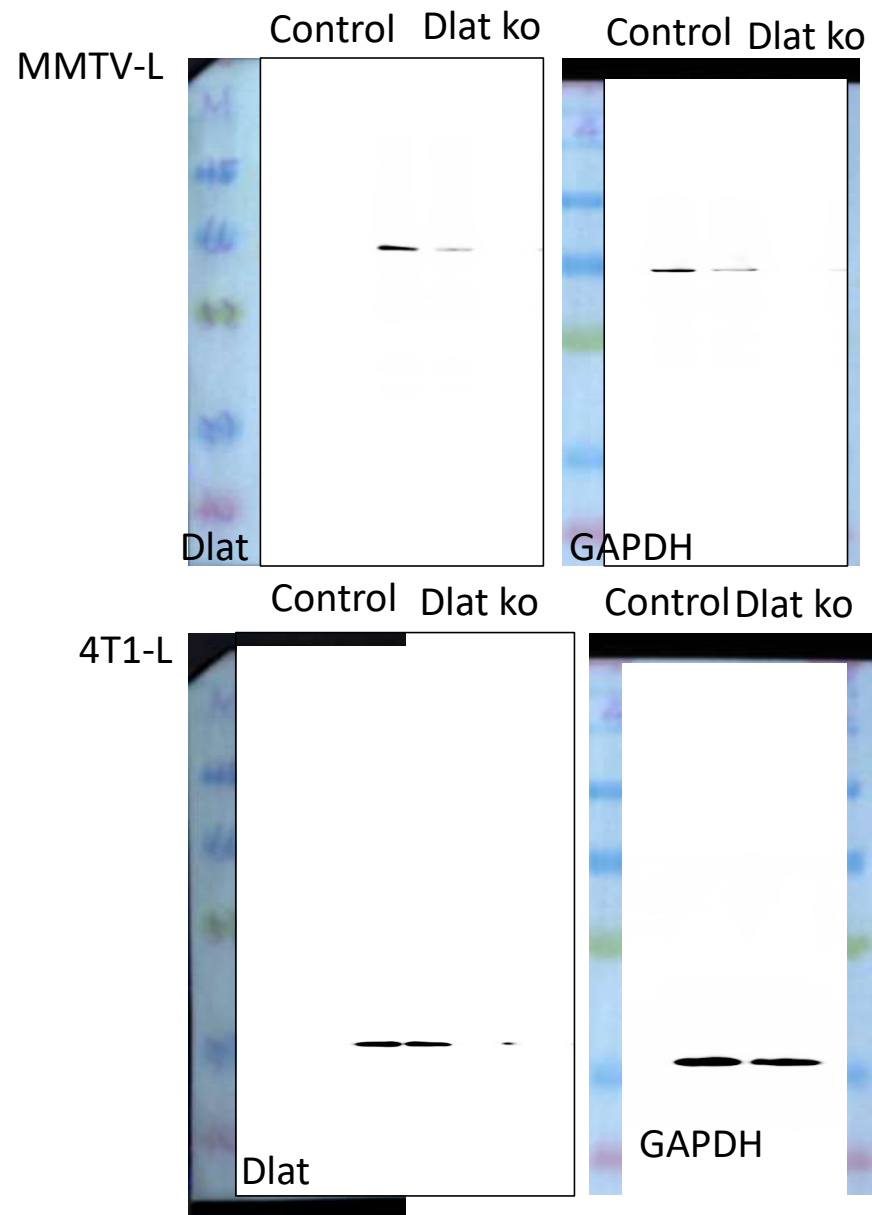

sFigure 3D

DLAT, proteintech, Cat.13426-1-AP  
GAPDH , MAB374

Supplement: Supplementary file 2 — Western blot [file 41419_2024_7205_MOESM2_ESM.pdf]
