# Supplementary material for: Metabolic shifts in lipid utilization and reciprocal interactions within the lung metastatic niche of triple-negative breast cancer revealed by spatial multi-omics
Source: Cell Death Dis. 2024 Dec 18;15(12):899. doi: 10.1038/s41419-024-07205-4 (PMC11655832; doi:10.1038/s41419-024-07205-4)
Supplement: Supplementary file 6 — Suppementary Table 6 [file 41419_2024_7205_MOESM6_ESM.docx]

sTable 6

| Cluster | Cell markers | ApoE | Soluble factors | Ki67 | Cell type |
| --- | --- | --- | --- | --- | --- |
| 18 | EPCAM+ | +++ | S100A6  S100A10 (+++)  S100A4 (++)  Gal-1 (M)  Gal-3 (-)  Spp1 (+-)  Thbs1 (+) | +- | Cancer cells |
| 19 | EPCAM+ | +++ | S100A4 (+-)  S100A10 (+++)  Spp-1 (++)  Gal-1 (+-)  Gal-3 (+-)  Thbs1 (+++) | ++ | Cancer cells |
| 7 | EPCAM+ | ++ | Gal-1(+++)  Gal-3(+-) |  | Cancer cells |
| 12 | CD45+CD68+CD163+ | - | Gal-3(+) |  | M2 macrophage |
| 17 | CD45+CD68+CD163+ | +++ | Gal-1 (+++) |  | M2 macrophage |
| 25 | CD45+CD163+ | +++ | SA100A6  S100A10  S100A4 (L)  Gal-1 (M)  Gal-3 (-)  Spp1 (L)  Thbs1+ | +- | M2 macrophage |
| 21 | CD163+ | + | S100A10 |  | M2 macrophage |
| 2 | CD45+CD11b+CD68^low^ | +- | S100A4 (+-)  S100A6 (+-)  Spp1 (+--)  Thbs1 (+) | + | monocyte |
| 6 | CD45+CD11b+ | +- | Spp-1 (+) |  | Immune cells |
| 13 | Ly6g+ | +- | Retnal (+)  Spp-1 (+)  Gal-1 (+) | ++ | Neutrophil |
| 23 | CD31+ | +++ | S100A4  S100A6  S100A10(+++) | +- | Endothelial cells |
| 24 | CD31+ | +++ | S100A10 (+++) |  | Endothelial cells |
| 15 | CD31+ | +- | Thbs1 | ++ | Endothelial cells |
| 16 | Cd31 | ++ | Thbs1 | ++ | Endothelial cells |
| 10 | Cd31+ | +- | Gal-1 | + | Endothelial cells |
| 22 | CD31+ | +- | Gal-3+++  Gal-1+ |  | Endothelial cells |
| 8 | EPCAM | +- |  |  | Normal cell |
| 3 | CD45+ | +- | S100A4 (+)  Thbs1(+++) | ++ | Immune cells |
| 11 | CD45+ | +- | Gal-3 (+++)  Spp-1(+-) | - | Immune cells |
| 14 | CD45+ | +- | Gal-1(+)  Gal-3(+-) |  | Immune cells |
|  |  |  |  |  |  |
